# Quantifying the effects of air pollution on respiratory ill health treated in primary care when the locations of the populations at risk are partially unknown

**DOI:** 10.1177/09622802261439259

**Published:** 2026-04-24

**Authors:** Qiangqiang Zhu, Duncan Lee, Oliver Stoner

**Affiliations:** 1 School of Mathematics and Statistics, University of Glasgow, UK

**Keywords:** Air pollution, Bayesian spatio-temporal model, neighbourhood matrix, respiratory prescribing rates in primary care

## Abstract

Most air pollution and health studies focus on severe outcomes such as hospitalisations and deaths, overlooking the impact that air pollution may have on non-hospitalised respiratory ill health treated in primary care. This paper presents a new study investigating the effects of NO_2_, PM_10_ and PM ${}_{2.5}$ on the prescription rates of respiratory medications in Scotland between 2016 and 2020 at a monthly resolution. To enhance the spatial accuracy of the exposure estimates, air pollution predictions at a 1 km ${}^{2}$ resolution are realigned to General Practioner (GP) surgery patient populations by accounting for where patients are likely to live rather than just where the GP surgery is. A Bayesian spatio-temporal conditional autoregressive model is utilised to account for spatial and temporal dependencies in the data, and this paper proposes two novel spatial neighbourhood matrices to better represent the spatial closeness among the patient populations registered at each GP surgery. These matrices improve model performance in capturing spatial correlation compared to standard distance-based approaches, such as using K -nearest neighbours approach. The results of the study suggest that particulate matter pollution has a significant impact on prescription rates for inhaled corticosteroids that are taken to prevent the symptoms of respiratory ill health, while NO_2_ demonstrates no such association.

## 1. Introduction

Air pollution is a complex mixture of numerous components, including gases such as nitrogen dioxide (NO_2_), and particulate matter which is commonly classified as particles ≤ 10 μ m (PM_10_) or 2.5 μ m (PM ${}_{2.5}$ ) in aerodynamic diameter. These and other air pollutants are some of the most important environmental risk factors for non-communicable diseases, affecting global health and well-being. In 2019, ambient air pollution was estimated to have caused 4.2 million premature deaths worldwide.^1^ In the UK it remains the leading environmental risk to public health, contributing to between 28,000 and 36,000 deaths per year.^2^ The financial burden is also substantial, with air pollution expected to cost the National Health Service and social care system £1.6 billion between 2017 and 2025.^3^ Research shows that long-term exposure to air pollutants can affect lung function, trigger asthma and chronic obstructive pulmonary disease (COPD) exacerbations, and increase the risk of lung cancer, imposing a considerable social and economic burden.^4–6^ The UK has one of the highest asthma prevalence rates globally, with approximately 5.4 million people receiving treatment.^7^ Additionally, COPD affects around 3 million individuals in the UK and accounts for approximately 1.4 million general practice (GP) consultations per year.^8^

Most of the epidemiological research on air pollution and health has focused on severe health outcomes, such as hospital admissions^9–11^ and mortalities.^12^ Large international collaborative studies have examined these severe end points, including the World Health Organisation-led Estimating the Morbidity from Air Pollution and its Economic Costs (EMAPEC) and Health Risks of Air Pollution in Europe (HRAPIE-2) projects. However, this almost exclusive focus on severe disease cases may underestimate the full burden of air pollution, because it overlooks its effects on non-hospitalised illness treated in primary care where disease cases with relatively mild symptoms are managed. An exception is Blangiardo et al.,^13^ who linked NO_2_ concentrations to respiratory medication prescriptions issued in general practice (GP, doctors) surgeries in England, using prescription rates as a proxy for the prevalence of respiratory illness not requiring hospitalisation. They found a very small association between NO_2_ and GP prescribing rates, with an estimated increase in prescribing rates of 0.07% when NO_2_ increased by 10 μ g/m ${}^{3}$ . In a later study, Lee^14^ separately modelled data on medications that relieve (i.e. short-acting $\beta_{2}$ agonists) and prevent (i.e. corticosteroids) the symptoms of respiratory ill health in Scotland, and found that increased concentrations of both PM_10_ and PM ${}_{2.5}$ by 2 μ g/m ${}^{3}$ were significantly associated with around 1% to 3% increases in prescribing rates.

These studies, although valuable, are not very recent and have a short temporal duration, which highlights the need for updated epidemiological evidence to inform current public health policies. For example, Blangiardo et al.^13^ analysed prescribing data from August 2010 to November 2012 in England, while Lee^14^ used data from October 2015 to August 2016 in Scotland. Both studies are at the population level, and have the drawback that they relate prescription rates for each GP surgery to estimate pollution concentrations at or near the GP surgery location. They are thus likely to suffer from exposure measurement error, due to the spatial misalignment between each GP surgery’s location and where its registered patient population actually live. Blangiardo et al.^13^ estimated air pollution concentrations at the GP surgery location, while Lee^14^ estimated average concentrations in arbitrary circular buffer zones around each surgery, both of which assume that all patients live very close to their GP surgery which is unrealistic. Furthermore, these studies utilised standard distance-based spatial neighbourhood matrices to model the residual spatial correlation structure in the GP-level prescribing data, which only took account of the distances between each pair of GP surgeries. This again may not accurately reflect the real-world spatial proximity and interactions between patients attending different GP surgeries, as patients do not live at the surgery itself and may not even attend their nearest surgery.

Therefore, this paper provides two novel contributions to the literature that addresses these limitations. Firstly, it presents a new Scotland-wide study quantifying the effects of air pollution on the prescribing rates of respiratory medications in primary (non-hospitalised) care, using more recent and long-term data from 2016 to 2020. We focus on the effects that NO_2_, PM_10_ and PM ${}_{2.5}$ have on prescription rates for medications that relieve and prevent the symptoms of respiratory disease such as asthma and COPD. Additionally, we compare the results from the pre-pandemic period (2016–2019) and the full study period (2016–2020), allowing us to examine whether pandemic-related disruptions altered the association between air pollution and respiratory prescription rates. Secondly, we propose novel binary neighbourhood matrices to capture the spatial adjacencies between the GP surgery patient populations, which are used in conjunction with conditional autoregressive (CAR)^15^ models to account for the residual spatial correlations present in the data. We show that these new adjacency structures better account for the spatial correlation in the data compared to simpler distance-decay based structures that only account for the GP surgery locations and not where their registered patients actually live. In a similar vein we enhance the spatial accuracy of the air pollution exposure estimates compared to the above studies, by again developing estimates based on where each surgery’s patient population are likely to live.

These new methodological developments are set within the Bayesian spatio-temporal random effects model proposed by Rushworth et al.,^16^ which is fitted using an integrated nested Laplace approximation (INLA) approach^17^ with variational Bayes approximations. Full details of our proposed methodology are given in Section 3, while the motivating study is presented in Section 2. Section 4 presents the results of the study, focusing on how the newly proposed neighbourhood matrices capture residual spatial autocorrelation, and the estimated effects of air pollution on chronic respiratory prescription rates. Finally, Section 5 summarises the study contributions and directions for future research.

## 2. Motivating study

**Figure 1.**
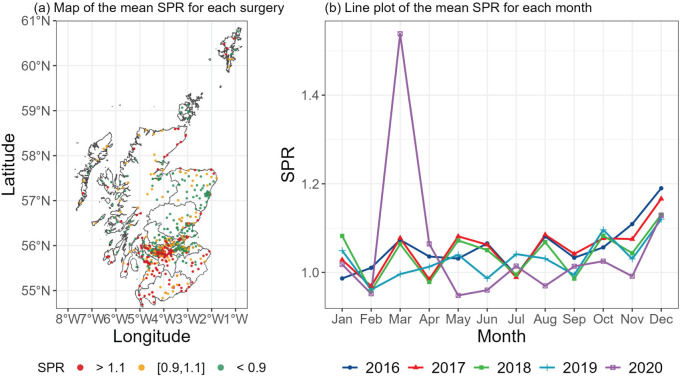
Spatial and temporal trends in the standardised prescription ratios for respiratory medications (the combined Both medication type) in Scotland. Panel (a) displays a map showing the spatial distribution of the mean SPR for each GP surgery between 2016 and 2020, while panel (b) displays a line plot showing the monthly variation in the mean SPRs over all GP surgeries in Scotland. For the former the SPR has been categorised into low (<0.9), medium ( [0.9,1.1] ) and high (>1.1) to aid clarity. SPR: standardised prescription ratio; GP: general practice.

The study is based in Scotland, UK, and utilises monthly data between 2016 and 2020. Our goal is to quantify the impact that air pollution exposure has on the prevalence of less severe cases of respiratory ill health that are treated in primary (non-hospitalised) care. In Scotland, primary care is provided by doctors who work in small groups co-located in GP surgeries, who are responsible for prescribing medications for alleviating and managing the symptoms of disease. Prescribing data were collected from a total of K=1006 distinct GP surgeries that were operational at some points between January 2016 and December 2020, with 964 being present in 2016 compared to 928 in 2020. This decrease is a result of the trend for surgeries to merge into larger entities,^18^ and illustrates that the number of spatial data points change over the study period. Figure 1(a) presents the spatial distribution of the surgeries that were operational at any point during the study, and shows that most surgeries are located within the central belt of Scotland containing Glasgow in the west and Edinburgh in the east.

### 2.1. Prescription data

In this study, we use the number of respiratory-related medications prescribed by each GP surgery as a proxy measure for the prevalence of less severe respiratory ill health that does not require hospitalisation. Monthly prescription counts for asthma and COPD medications were collected from the Prescribing Information System database of Public Health Scotland, including medications prescribed for both relieving and preventing the symptoms of these diseases. Following Blangiardo et al.^13^ and Lee,^14^ these medications comprise bronchodilators (see British National Formulary (BNF) Chapter 3.1) that relieve the symptoms of respiratory ill health (hereafter Reliever) and inhaled corticosteroids (BNF Chapter 3.2) that prevent the symptoms of respiratory ill health (hereafter Preventer). Additionally, we also consider counts of both medication types jointly (hereafter Both) to assess the sensitivity of our results. To adjust for demographic differences across GP surgeries, expected prescription counts were calculated using indirect standardisation. Specifically, the numbers of male and female patients registered at each surgery in age groups (0–4, 5–14, 15–24, 24–44, 45–64, 65–74, 75–84 and 85+) were multiplied by national age-sex specific prevalence rates of asthma and COPD from the Scottish Primary Care Information Resource database (see Section S1.1 of the Supplemental materials), and then summed to estimate the expected numbers of patients living with respiratory disease at each surgery. These expected numbers were then rescaled to ensure that the total observed and expected prescription counts across all spatio-temporal units are equal.

The standardised prescription ratio (SPR) for each GP surgery and month is computed by dividing the observed prescription count by the expected count, and is analogous to computing the standardised mortality ratio when modelling death data. A value above one indicates a higher risk of respiratory disease compared to the national average, with an SPR of 1.2 corresponding to a 20% increased risk. The spatial distribution of the SPRs for the combined Both medication type averaged between January 2016 and December 2020 is depicted in Figure 1(a), which categorises them into three groups for reasons of clarity in the figure: below average (<0.9); around average ( [0.9,1.1] ); and above average (>1.1). Areas in Glasgow, southern Scotland, and along some parts of the coast typically exhibit higher SPRs, while Edinburgh and Aberdeen stand out as having lower risks. The monthly temporal trend in the mean SPR (again the Both medication type) across all GP surgeries is displayed in Figure 1(b), which generally exhibits somewhat random fluctuations with a little apparent trend. The exceptions to this are the peaks in SPRs in December each year and in March 2020, which is likely to be because patients stock up on medications while their GP surgeries are closed during the Christmas vacation and again following the national lockdown at the start of the Covid-19 pandemic. The spatial and temporal trends for the Preventer and Reliever medication types are broadly similar and are not shown for brevity.

### 2.2. Air pollution data

The geographical region and monthly temporal resolution of our study aligns with the spatio-temporal resolution used in a recent air pollution prediction study by Zhu et al.^19^ The aim of this work is to compare the predictive accuracy of a number of different methodologies, including simple linear and additive models, spatio-temporal smoothing models and machine learning algorithms. Each model was applied to the set of monthly average pollution concentrations measured by a network of fixed-site monitors across Scotland, which numbers 94 (NO_2_), 93 (PM_10_) and 79 (PM ${}_{2.5}$ ) sites in total across the 60-month study period. The covariates used in these models included numerical pollution and meteorological model outputs at a 1 km ${}^{2}$ resolution, as well as other covariates quantifying population density and distance to the nearest road. The best-performing model for each pollutant was assessed by quantifying its out-of-sample predictive performance, which was achieved by splitting the measurement data into an 80% training set and a 20% test set by site. Each model was fitted to the training set (tuning parameters were optimised via a 10-fold cross validation procedure applied to this training set), before being used to predict observations in the test set. This process was repeated 10 times to ensure that the results were not affected by a single choice of training and test split.

The accuracy of the point predictions were measured by their root mean squared error (RMSE) and median absolute error (MAE), while the appropriateness of their 95% prediction intervals was assessed by their coverage proportions and average interval widths. This model comparison resulted in a random forest being the best prediction algorithm for both NO_2_ and PM ${}_{2.5}$ , while a simple linear model performed best for PM_10_. The RMSE and MAE values from these best-performing models are: NO_2_: RMSE=7.70 , MAE=4.36 ; PM_10_: RMSE=2.73 , MAE=1.44 ; and PM ${}_{2.5}$ : RMSE=1.15 , MAE=0.54 ; which compares to the original monthly average measurement data that have the following ranges: NO_2_: [1.1, 76.5]; PM_10_: [2.8, 38.5]; PM ${}_{2.5}$ : [1.3, 23.9]. The above-mentioned best-performing models were subsequently used to predict monthly average concentrations on a 1 km ${}^{2}$ grid covering all of Scotland for all 60 months, which we use here as the basis for our air pollution exposure metrics. Further details on these predictions are given in Zhu et al.^19^

### 2.3. Confounders

Data on a number of important confounding factors were collected for this study, the first of which is socio-economic deprivation that strongly correlates with the risk of respiratory disease.^14,20^ In Scotland, socio-economic deprivation is quantified using the Scottish Index of Multiple Deprivation (SIMD),^21^ and here we use the index for 2020. The index includes indicators in seven domains such as income, employment, education, health, access to services, crime, and housing, and it is constructed for 6976 small non-overlapping areal units called data zones (DZs). The indicators in the health domain were removed to avoid circularity, that is, using health indicators to model health outcomes. Additionally, some pairs of SIMD indicators show high correlations, so only a subset of the SIMD indicators were selected in the models. Further details are provided in Section S1.2 of the Supplemental materials.

Epidemiological studies have consistently found that both extreme heat and cold are linked to increased respiratory morbidity and mortality in asthma and COPD cases.^22–25^ Although humidity is not typically considered a health risk factor, high humidity can promote mould growth, producing spores that, when inhaled, can trigger respiratory symptoms such as coughing and wheezing, particularly in individuals with mould allergies.^26^ This can exacerbate asthma and COPD symptoms and pose increasing risks for patients.^11,27^ Therefore, in this study we include temperature and humidity as meteorological confounders in the model. These monthly data were collected from the HadUK-Grid data sets^28^ at a 1 km ${}^{2}$ grid square resolution over Scotland.

Disparities in the prevalence and severity of asthma and COPD by ethnicity have been identified,^29,30^ so we obtained ethnicity data from the 2022 Scottish census (the closest year of data available). For each DZ the ethnicity data include the percentage of the population belonging to the following ethnic groups: (i) White; (ii) South Asian (Indian/Pakistani/Bangladeshi) descent; (iii) Chinese descent; and (iv) Black. The percentages in each of these groups sum to nearly 100 for most data zones, inducing collinearity between these four covariates. Therefore, the percentage White variable is not included in the analysis. The correlations among the remaining three ethnic groups (Chinese, South Asian, and Black) are relatively low, ranging between 0.25 and 0.29, and are hence included as covariates in the model.

### 2.4. Spatial realignment

The study consists of data for t=1,…,T=60 consecutive months between January 2016 and December 2020, and collectively the prescription, air pollution and covariate data relate to three different spatial scales. Prescription data were recorded at k=1,…,K=1006 GP surgeries, but recall that not all surgeries were operational for all 60 months, leading to different numbers of spatial data points for each month. The k th GP surgery is located at ${\text{s}}_{k}$ , while the spatial extent of its patient catchment area is denoted by $S_{k}$ . Patients do not necessarily attend their nearest GP surgery, which means that the set of patient catchment areas $\left\{S_{1},\ldots ,S_{k}\right\}$ constitute a set of overlapping areal units. The spatial extent of catchment area $S_{k}$ is partially unknown, because the only information available to quantify it measures its population intersections with each data zone. Here, we denote the m=1,…,M=6976 data zones by $\left\{A_{1},\ldots ,A_{M}\right\}$ . The specific population intersection data provided by Public Health Scotland are denoted by $\left\{p_{t}(A_{m}\cap S_{k})\right\}$ , which quantifies the number of people who live in data zone $A_{m}$ and are registered at the k th GP surgery in month t . Note, each person can only be registered at one GP surgery. Thus, while these data provide some information on the spatial extents of the GP surgery catchment areas, they do not give an exact geographical boundary as we do not know where in each data zone each patient lives.

The SIMD and ethnicity confounders are available at the data zone scale, while the air pollution concentrations and the meteorological confounders are available at a third spatial scale, namely the set of i=1,…,I=4,708,065 1 km ${}^{2}$ grid squares across Scotland denoted here by $\left\{H_{1},\ldots ,H_{I}\right\}$ . As these grid squares and the data zones have fixed geographical boundaries, we computed their areas of intersection, which for grid square $H_{i}$ and data zone $A_{m}$ is denoted by $a(H_{i}\cap A_{m})$ . Therefore, the first statistical challenge we face when modelling these data is the *spatial change of support problem*, as we need all the variables to be on a common spatial scale. The spatial scale used for inference is the GP surgery scale, because that is the scale at which the disease count data are available. Thus, any covariate at the DZ scale $\left\{x_{t}(A_{m})\right\}$ was spatially realigned to the GP scale $\left\{x_{t}(S_{k})\right\}$ by population weighted averaging, that is, by computing

$$x_{t}(S_{k})=\sum_{m=1}^{M}\frac{p_{t}(A_{m}\cap S_{k})}{\sum_{r=1}^{M}p_{t}(A_{r}\cap S_{k})}x_{t}(A_{m})$$

Note, the population intersect data $\left\{p_{t}(A_{m}\cap S_{k})\right\}$ are only available on an annual basis, so we assume that these population intersections apply for all months within the year. Finally, the 1 km ${}^{2}$ gridded pollution exposures and meteorological confounders were spatially rescaled to the DZ scale and then subsequently to the GP scale. The DZ to GP scale realignment is achieved using the population-weighted averaging outlined above, while the grid square to DZ realignment is achieved using area-weighted averaging via

$$x_{t}(A_{m})=\sum_{i=1}^{I}\frac{a(H_{i}\cap A_{m})}{\sum_{j=1}^{I}a(H_{j}\cap A_{m})}x_{t}(H_{i})$$

### 2.5. Exploratory analysis

We fitted overdispersed negative binomial count data models to six separate prescription data sets, which include the three different medication types (reliever, preventer and both) for two time periods: (i) the full study period from 2016 to 2020; and (ii) the pre-pandemic period from 2016 to 2019 to remove any pandemic effects. Each model includes the expected prescription counts as an offset term as well as the set of potential confounders described in Section 2.3. To check for the presence of multicollinearity, we computed the variance inflation factors (VIFs) for this covariate set, and sequentially removed covariates that had VIF values above 5, which is a commonly used rule of thumb for when collinearity is likely to adversely affect the model results. This procedure was applied separately for each data set, and resulted in the removal of six confounders in all cases (see Section S1.2 of the Supplemental materials). The confounders removed include those summarising employment deprivation and no school qualifications (correlated with income deprivation), housing overcrowding (correlated with no central heating in housing) and public transport travel times to a post office, GP surgery and retail centre (correlated with the drive time equivalents).

Upon selecting this confounder set we then added a single pollutant to each model, as well as a set of temporal trend terms. The latter includes year (5 levels) and month-of-year (12 levels) indicator variables, together with an additional indicator for March 2020. This specification adjusts for year to year temporal variation and seasonality, while explicitly allowing for the anomalous spike in prescriptions observed in the first month of the Covid-19 lockdown (see Figure 1) that was likely due to people stocking up on medications due to the uncertainty surrounding the national lockdown that was introduced on 24th March 2020. Note, the data do not support the addition of a longer-term Covid effect indicator for the months succeeding March 2020, which is probably due to the fact that after stocking up in March there was no need to stock up on further medications again in subsequent months.

We then examined the presence of spatial correlation in the residuals using Moran’s I statistic,^31^ where a binary neighbourhood matrix based on the five nearest neighbours method (Euclidean distances were calculated between the GP surgery locations $\left\{{\text{s}}_{k}\right\}$ ) was used to compute the indicator. Similarly, residual temporal correlation was examined using the lag 1 autocorrelation function. The results of this correlation analysis presented below relate to when NO_2_ is the pollutant included in the model, but the results for the remaining pollutants are analogous and are not shown for brevity. We found that for each of the six prescription data sets all of the 60 months exhibit significant positive residual spatial correlation at the 5% level, indicating that spatial correlation is pervasive and needs to be accounted for. Additionally, residual temporal correlation is also consistently present, with between 23.05% and 27.31% of the GP surgeries exhibiting significant temporal correlation at lag 1 across the different medication types.

## 3. Methodology

This section proposes a novel Bayesian hierarchical spatio-temporal model for quantifying the effects of air pollution on GP prescribing rates, where the novel contribution concerns how to construct a spatial neighbourhood matrix for capturing the spatial closeness between the patient populations registered at each pair of GP surgeries. Inference for this model is based on INLAs with variational Bayes approximations, using the R package INLA.^17^ The model is presented in stages below, beginning with the data likelihood component.

### 3.1. Data likelihood model

Letting k=1,…,K=1006 index the complete set of GP surgeries that appears at least once throughout the t=1,…,T=60 -month study period, the negative binomial likelihood model is given by

(1) $$\begin{array}{rl} Y_{t}(S_{k}) & ∼\text{Negative-Binomial}\left(e_{t}(S_{k})\theta_{t}(S_{k}),\zeta \right)\;\text{for }k\in S^{\left(t\right)}\;\text{and}\;t=1,\ldots ,T,\\ \ln\;\left(\theta_{t}(S_{k})\right) & ={\text{z}}_{t}(S_{k}{)}^{⊤}\delta +x_{t}(S_{k})\beta +\phi_{t}(S_{k}) \end{array}$$

As previously described, not all GP surgeries are operational at each month of the study, and hence $S^{\left(t\right)}\subset \{1,\ldots ,K\}$ denotes the set of indices for the GP surgeries that are operational in month t . The response variable $Y_{t}(S_{k})$ denotes a count of the number of prescriptions issued by the GP surgery with catchment area $S_{k}$ in month t , while $e_{t}(S_{k})$ denotes the indirectly standardised expected count (accounting for demographics) that is included in the model as a fixed offset. The expected value of this distribution is the product of the expected count $e_{t}(S_{k})$ and the (relative) risk of disease $\theta_{t}(S_{k})$ , while the hyperparameter ζ is the overdispersion parameter of the negative binomial distribution. Here, log(ζ) is assigned a penalised complexity Gamma prior distribution (pc.mgamma, default in INLA software), which is chosen to be weakly informative and hence let the data inform on the level of overdispersion. The risk of disease is modelled on the natural log scale by a p×1 vector of confounders ${\text{z}}_{t}(S_{k})$ , the pollution exposure $x_{t}(S_{k})$ , and a spatio-temporal random effect $\phi_{t}(S_{k})$ to allow for residual spatio-temporal correlation. The regression parameters for the confounders ( $\delta =(\delta_{1},\ldots ,\delta_{p})$ ) and the pollution exposure ( β ) are assigned weakly informative independent Gaussian priors with mean 0 and precision 0.001, again to let the data inform on their value. Finally, we only incorporate a single pollutant in each model run, due to the collinearity between the three pollutants considered in this study.

### 3.2. Spatio-temporal random effects model

A range of prior distributions have been proposed for modelling the correlations among the spatio-temporal random effects $\left\{\phi_{t}(S_{k})\right\}$ , and in this analysis we utilise the multivariate first-order autoregressive process model with CAR spatial structure proposed by Rushworth et al.^16^ The model is expressed by

(2) $$\begin{array}{rl} \phi_{1} & ∼\text{N}(0,[\tau \text{Q}(\text{W},\rho ){]}^{-1}),\\ \phi_{t}|\phi_{t-1} & ∼\text{N}(\alpha \phi_{t-1},[\tau \text{Q}(\text{W},\rho ){]}^{-1})\;\text{for }t=2,\ldots ,T \end{array}$$

where $\phi_{t}=(\phi_{t}(S_{1}),\ldots ,\phi_{t}(S_{K}))$ denotes the complete set of spatial random effects for all K GP surgeries during month t . Thus, $\phi_{t}$ has the same length for all months t , and Q(W,ρ) is a K×K time-invariant precision matrix. For any given month where a particular GP surgery is not operational, its corresponding data values from (1) are treated as missing values. Temporal correlation is captured through the mean via $\alpha \phi_{t-1}$ , while spatial correlation is captured through the covariance structure via $[\tau \text{Q}(\text{W},\rho ){]}^{-1}$ . The parameters (α,ρ) control the level of temporal ( α ) and spatial ( ρ ) correlation among the random effects, with values of zero corresponding to independence in time and space, while values close to one correspond to strong positive correlation. The spatial dependence parameter ρ∈[0,1] is assigned a weakly informative Gaussian prior on the logit scale, that is, log(ρ/(1−ρ))∼N(0,2) . Similarly, the temporal dependence parameter α∈[−1,1] is assigned a weakly informative Gaussian prior on the transformed scale, log((1+α)/(1−α))∼N(0.25,2) . The level of uncertainty in the random effects is controlled by the precision parameter τ , which is assigned a weakly informative half-normal prior on the standard deviation scale, that is, $\sigma =1/\sqrt{\tau }∼\text{Half-Normal}(0,\text{sd}=1.5)$ , following the recommendation by Gelman.^32^ Further details about these prior specifications are provided in Section S2 of the Supplemental materials.

The spatial correlation structure in the data is modelled by the CAR prior proposed by Leroux et al. (2000),^33^ which has the precision matrix

(3) Q(W,ρ)=ρ(diag(W1)−W)+(1−ρ)I

where 1 denotes a K×1 vector of ones while I denotes a K×K identity matrix. The spatial closeness between each pair of GP surgeries is captured by the non-negative K×K neighbourhood matrix W , where typically $w_{kj}>0$ if the patient populations represented by surgeries $\left(S_{k},S_{j}\right)$ are close together, and $w_{kj}=0$ otherwise ( $w_{kk}=0\;\forall \;k$ ). A binary specification is commonly assumed for this matrix, because it encourages sparsity in Q(W,ρ) and hence fast model fitting. Under either a binary or a non-binary construction, multivariate Gaussian theory shows that the partial correlations implied by the Leroux CAR model are given by

$$\text{Corr}\left(\phi_{t}(S_{k}),\phi_{t}(S_{j})|\phi_{t}(-S_{k},S_{j})\right)=\frac{\rho w_{kj}}{\sqrt{\left(\rho \sum_{i=1}^{n}w_{ki}+1-\rho )(\rho \sum_{l=1}^{n}w_{jl}+1-\rho \right)}}$$

where $\phi_{t}(-S_{k},S_{j})$ denotes all the random effects at time period t except $\left\{\phi_{t}(S_{k}),\phi_{t}(S_{j})\right\}$ . Thus, if $w_{kj}=0$ then $\left\{\phi_{t}(S_{k}),\phi_{t}(S_{j})\right\}$ are modelled as conditionally independent given the remaining random effects at time t , while if $w_{kj}>0$ then they are modelled as marginally correlated.

### 3.3. Neighbourhood matrix

The novel methodological contribution of this paper is how to construct the spatial neighbourhood matrix W for use in the spatio-temporal CAR model, which is crucial because the previous equation (3) shows that it controls the spatial correlation structure assumed for the random effects $\left\{\phi_{t}\right\}$ . The most common approach to defining W in spatial areal unit data is through the border sharing rule, which specifies a binary neighbourhood matrix whose values are determined by whether or not each pair of areal units shares a common border. However, the GP surgery catchment areas overlap and have partially unknown spatial extents, so this approach is not feasible. The second most common approach is to use a D nearest neighbours structure, where $w_{kj}=1$ if the j th GP surgery location ${\text{s}}_{j}$ is one of the D nearest neighbours of the k th GP surgery location ${\text{s}}_{k}$ , and is zero otherwise. This matrix is then made symmetric by setting $w_{jk}=1$ if under this construction $\left\{w_{kj}=1,w_{jk}=0\right\}$ . The choice of D in this setting is somewhat arbitrary, and typically values around D=5 are chosen as this tends to match the average numbers of neighbours each areal unit would have under the commonly used border sharing rule. In the present context this D nearest neighbours construction would define the spatial correlation structure based solely on where the GP surgeries are located, rather than on where their patient populations live and the extent to which they overlap and interact. As it is the patient populations rather than the GP surgery buildings that have respiratory disease and use the medications, this construction seems inappropriate.

Therefore, here we propose two novel binary specifications for W denoted by ${\text{W}}^{\left(O\right)}$ and ${\text{W}}^{\left(S\right)}$ , which account for the extent to which the patient populations from two GP surgeries overlap and are spatially close together. The only data available to quantify these population overlaps are at the data zone level, and comprise population counts $\left\{p_{t}(A_{m}\cap S_{k})\right\}$ defined earlier. The first matrix ${\text{W}}^{\left(O\right)}$ (‘O’ for overlap) only considers direct population overlaps, that is, the extent to which the patient populations from two GP surgeries live in the same data zones. For the GP surgeries with catchment areas $\left(S_{k},S_{j}\right)$ the kj th element of the matrix is based on

(4) $$w_{kj}^{*(O)}=\sum_{m=1}^{M}\left(\frac{1}{T_{k}}\sum_{t:p_{t}(S_{k})>0}\frac{p_{t}(A_{m}\cap S_{k})}{p_{t}(S_{k})}\times \frac{1}{T_{j}}\sum_{t:P_{t}(S_{j})>0}\frac{p_{t}(A_{m}\cap S_{j})}{p_{t}(S_{j})}\right)$$

where $P_{t}(S_{k})=\sum_{r=1}^{M}p_{t}(A_{r}\cap S_{k})$ denotes the total size of the patient population for the k th GP surgery. Additionally, $T_{k}$ and $T_{j}$ denote the number of time periods that GP surgeries ${\text{s}}_{k}$ and ${\text{s}}_{j}$ are operational. This construction first computes the product of the (time-averaged) proportions of the two GP surgery’s patient populations who live in the same data zone $A_{m}$ , before summing these products over all data zones in Scotland. Note, most of these time-averaged products will be zero. Thus, two GP surgeries will have a higher value of $w_{kj}^{*(O)}$ if their populations are generally located in the same data zones, and lower values if they are distributed across different data zones. In the extreme, if the patient populations from the GP surgeries located at $\left\{{\text{s}}_{k},{\text{s}}_{j}\right\}$ are both all located in the same data zone for all time periods, then $w_{kj}^{*(O)}=1$ . Conversely, if they have no data zone level population overlaps then $w_{kj}^{*(O)}=0$ . The above construction only considers patient population overlaps in the same data zone, and thus our second novel specification ${\text{W}}^{\left(S\right)}$ (‘S’ for spatial) considers population overlaps in the same and spatially neighbouring data zones. Here, spatially neighbouring means that two data zones share a common border. This construction naturally extends (4) and is given by

(5) $$w_{kj}^{*(S)}=\sum_{m=1}^{M}\left(\frac{1}{T_{k}}\sum_{t:p_{t}(S_{k})>0}\sum_{i\in R_{m}}\frac{p_{t}(A_{i}\cap S_{k})}{p_{t}(S_{k})}\times \frac{1}{T_{j}}\sum_{t:p_{t}(S_{j})>0}\sum_{i\in R_{m}}\frac{p_{t}(A_{i}\cap S_{j})}{p_{t}(S_{j})}\right)$$

where $R_{m}$ denotes the set of indices that include the target data zone $A_{m}$ , along with all data zones that share a border with $A_{m}$ . Thus, population overlaps between two GP surgeries are aggregated over each individual data zone and its spatial neighbours, giving a more comprehensive picture of the spatial closeness between two GP surgery’s patient populations.

The constructions $\left(w_{kj}^{*(O)},w_{kj}^{*(S)}\right)$ are inherently non-binary, and also contain a large number of very small non-zero values corresponding to two GP surgeries that only have a small number of patients living in the same or neighbouring data zones. These very small non-zero values may not be representative of the closeness of the two surgeries in question, and will lead to a less sparse neighbourhood matrix which will increase the model fitting time. Additionally, initial analyses showed that a binary matrix constructed based on $\left(w_{kj}^{*(O)},w_{kj}^{*(S)}\right)$ performed much better than a non-binary one in the motivating study (see the next section for details), which is likely to be due to a number of reasons. Firstly, a non-binary matrix would lead to some of the row sums $\sum_{j=1}^{K}w_{kj}^{*(O)}$ being very small (minimum = 0.0001, mean = 0.12, maximum = 0.997), and in CAR models this results in division by very small numbers in the mean and variance of the conditional distribution of $\left\{\phi_{t}(S_{k})|\phi_{t}(S_{1}),\ldots ,\phi_{t}(S_{k-1}),\phi_{t}(S_{k+1}),\ldots ,\phi_{t}(S_{K})\right\}$ , which would lead to numerical instability and unreliable smoothing. Additionally, the degree of spatial smoothing would be highly uneven, because some $\left(w_{kj}^{*(O)},w_{kj}^{*(S)}\right)$ values are close to one while others are close to zero, meaning that a few large weights dominate the smoothing process which will pull some random effects strongly towards only one neighbour while effectively ignoring the others. To address these limitations we create binary neighbourhood matrices based on $\left(w_{kj}^{*(O)},w_{kj}^{*(S)}\right)$ , and additionally apply a threshold ( ε or α ) so that very small values are set to zero. Specifically, we define the elements in the neighbourhood matrices $\left({\text{W}}^{\left(O\right)},{\text{W}}^{\left(S\right)}\right)$ as follows

(6) $$w_{kj}^{\left(O\right)}=\left\{ \begin{array}{ll} 1 & \text{if }w_{kj}^{*(O)}>\varepsilon \text{ and }k\neq j\\ 0 & \text{otherwise} \end{array}\right.,\;w_{kj}^{\left(S\right)}=\left\{ \begin{array}{ll} 1 & \text{if }w_{kj}^{*(S)}>\alpha \text{ and }k\neq j\\ 0 & \text{otherwise} \end{array}\right.$$

We consider a range of thresholds ε∈{0.0001,0.0005,0.001,0.005,0.01} and α∈{0.005,0.01,0.05,0.1,0.5} for the motivating study in the next section, and illustrate the impact of these choices on the number of neighbours (non-zero elements) for each GP surgery in Section S3 of the Supplemental materials. The optimal threshold is the one that minimises the levels of spatial correlation in the model residuals across the 60 months of the study, because the role of the neighbourhood matrix in conjunction with the random effects is to capture these correlations in the data.

### 3.4. Measuring residual spatial correlation

The level of spatial correlation in the residuals $\left\{r_{1t},\ldots ,r_{Kt}\right\}$ for each month t is measured by Moran’s I statistic,^31^ which, based on a generic neighbourhood matrix $\text{W}=\{w_{kj}\}$ , is given by

$$I_{t}=\frac{K\sum_{k=1}^{K}\sum_{j=1}^{K}w_{kj}(r_{kt}-{\bar{r}}_{t})(r_{jt}-{\bar{r}}_{t})}{\left(\sum_{k=1}^{K}\sum_{j=1}^{K}w_{kj}\right)\sum_{k=1}^{K}(r_{kt}-{\bar{r}}_{t}{)}^{2}}\;\in \;[-1,1]$$

where ${\bar{r}}_{t}=(1/K)\sum_{k=1}^{K}r_{kt}$ . A value of zero corresponds to spatial independence, while values respectively above and below zero correspond to positive and negative spatial correlation. A permutation-based hypothesis test (based on 10,000 random permutations of the data) can be constructed to test the significance of the residual spatial correlation, whose null hypothesis represents independence via $H_{0}:I_{t}=0$ . The alternative hypothesis could be $H_{1}:I_{t}>0$ or $H_{1}:I_{t}\neq 0$ , and while the former is the most obvious as data typically exhibit positive spatial correlation, spatial random effects models can over-smooth the data and induce negative spatial correlation into the residuals that can be identified by the latter hypothesis. Therefore, we measure the ability of each neighbourhood matrix at capturing the spatial correlation in the data by computing the following three metrics:

- *Positive spatial autocorrelation indicator (PSAI):* The percentage of the T months that have statistically significant Moran’s I values at the 5% level based on $H_{1}:I_{t}>0$ .
- *General spatial autocorrelation indicator (GSAI):* The percentage of the T months that have statistically significant Moran’s I values at the 5% level based on $H_{1}:I_{t}\neq 0$ .
- *Mean absolute Moran’s I (MAI):* The mean absolute value of Moran’s I over all T months computed by $\text{MAI}=(1/T)\sum_{t=1}^{T}|I_{t}|$ .

The first two of these metrics are used to assess how well each neighbourhood matrix has modelled the spatial correlation in the data (ideal values are 5%), while the last metric should be as small as possible and is used to pick the optimal threshold.

## 4. Results of the motivating study

The results of the study are presented below, and focus on the ability of the different neighbourhood matrices to capture the spatial correlation in the data (see Section 4.2), and the estimated effects of air pollution on prescribing rates for respiratory medications (see Section 4.3).

### 4.1. Study design

The study comprises 18 pollutant–outcome pairs, which are all possible combinations of three respiratory disease measures (preventer, reliever and both), three pollutants (NO_2_, PM_10_, PM ${}_{2.5}$ ), and two time periods (2016–2020 and 2016–2019). This dual study period allows us to assess the impact of the Covid-19 pandemic on the associations between air pollution and respiratory ill health. For each of these 18 analyses we fit the model with 11 different neighbourhood matrices, which includes one constructed using the five nearest neighbours method (hereafter ${\text{W}}^{\left(5nn\right)}$ ), as well as ${\text{W}}^{\left(O\right)}$ fitted separately with thresholds ε∈{0.0001,0.0005,0.001,0.005,0.01} , and ${\text{W}}^{\left(S\right)}$ fitted separately with thresholds α∈{0.005,0.01,0.05,0.1,0.5} .

### 4.2. Assessment of residual spatial correlation

**Figure 2.**
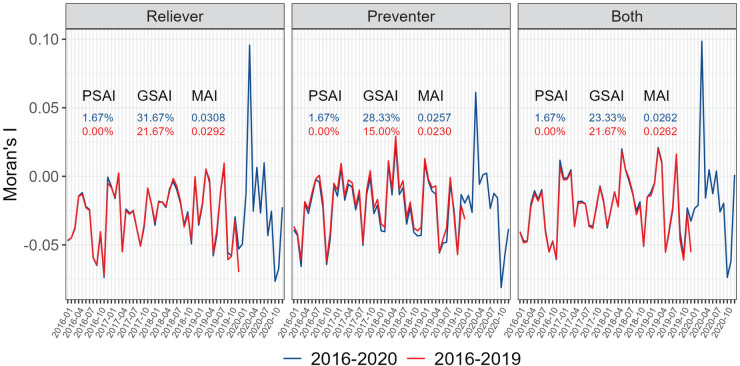
Moran’s I statistics quantifying the level of spatial correlation in the residuals for each month when using ${\text{W}}^{\left(5nn\right)}$ as the neighbourhood matrix. Each panel represents a different disease outcome, while red lines relate to the 2016–2019 study period while the blue lines relate to 2016–2020. PSAI, GSAI and MAI indicators are listed in each case. PSAI: positive spatial autocorrelation indicator; GSAI: general spatial autocorrelation indicator; MAI: mean absolute Moran’s I .

**Figure 3.**
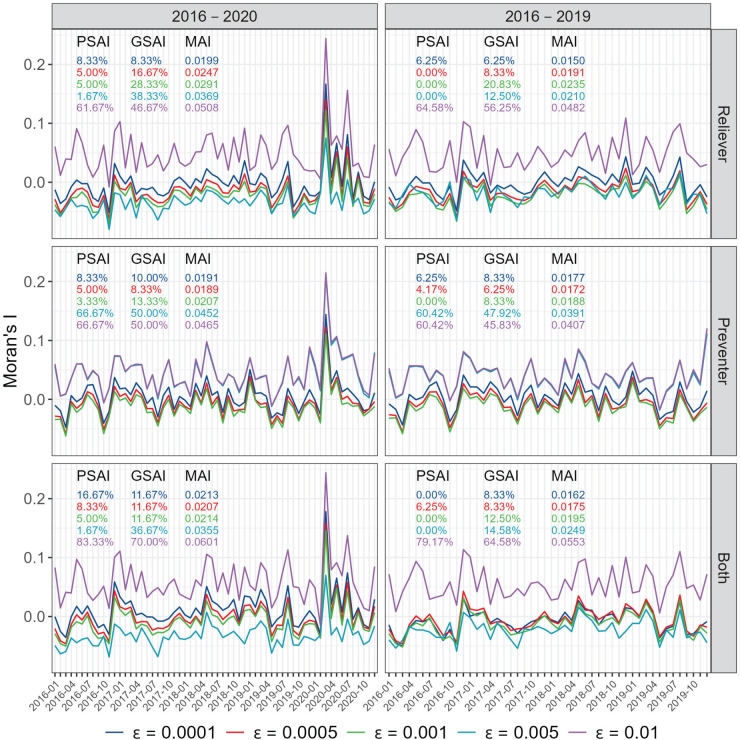
Moran’s I statistics quantifying the level of spatial correlation in the residuals for each month when using ${\text{W}}^{\left(O\right)}$ as the neighbourhood matrix. Each panel represents a different disease outcome and time period, while the coloured lines relate to the different thresholds ε . PSAI, GSAI and MAI indicators are listed in each case. PSAI: positive spatial autocorrelation indicator; GSAI: general spatial autocorrelation indicator; MAI: mean absolute Moran’s I .

Figures 2 and 3 present the levels of spatial correlation in the model residuals over all 60 months when using ${\text{W}}^{\left(5nn\right)}$ (Figure 2) and ${\text{W}}^{\left(O\right)}$ (Figure 3) for each medication type and study period. The corresponding plot for ${\text{W}}^{\left(S\right)}$ is provided in Section S4.1 of the Supplemental materials. These plots are based on the models fitted including NO_2_ as the single pollutant, and the results when including the other pollutants are almost identical and not shown for brevity. Each plot presents the Moran’s I statistic for each month of the study and for each threshold (for ${\text{W}}^{\left(O\right)}$ ), and the PSAI/GSAI/MAI metrics are given in each panel to summarise these residual correlations.

Figure 2 highlights that for both study periods (red: 2016–2019; blue: 2016–2020) ${\text{W}}^{\left(5nn\right)}$ fails to adequately capture the spatial correlation in all three medication types. The models typically induce negative spatial correlation into the residuals as a result of over-smoothing, which is evident because almost all of the Moran’s I values are negative. The exception to this is during the start of the Covid-19 pandemic in March 2020, where there is still positive residual correlation for all three medication types. The induced negative spatial correlations are generally statistically significant, as the GSAI indicator ranges between 15% and 32% across the medication types and study periods, when under independence it should be close to 5%. Conversely, the newly proposed binary neighbourhood matrices $\left\{{\text{W}}^{\left(O\right)},{\text{W}}^{\left(S\right)}\right\}$ are much better at capturing the spatial correlation in the data, because in both cases at least one threshold (ε,α) gives a GSAI value relatively close to 5% for each medication type and study period (see Figure 3 and Supplemental Figure S5).

For ${\text{W}}^{\left(O\right)}$ , small threshold values seem to provide the best model fit, with ε=0.0001 typically yielding the lowest MAI values between 0.0150 and 0.0213. In contrast, larger values of ε tend to either over or under smooth the data, resulting in either more positive or negative correlations in the residuals. For ${\text{W}}^{\left(S\right)}$ , the middle threshold values (e.g. α={0.01,0.05,0.1} ) seem to most appropriately capture the spatial correlation in the data, because they result in the smallest MAI values between 0.0153 and 0.0229 across all data sets. Therefore, ε=0.0001 and α=0.1 seem to be the optimal thresholds for ${\text{W}}^{\left(O\right)}$ and ${\text{W}}^{\left(S\right)}$ , because they typically minimise the GSAI and offer the lowest MAI values thus providing the best control for residual spatial correlation. Based on these optimal threshold ${\text{W}}^{\left(O\right)}$ and ${\text{W}}^{\left(S\right)}$ perform similarly, having similar GSAI, PSAI and MAI values. They both however provide a substantial improvement on ${\text{W}}^{\left(5nn\right)}$ , because their MAI values range between ( ${\text{W}}^{\left(O\right)}$ : 0.0150–0.0213) and ( ${\text{W}}^{\left(S\right)}$ : 0.0158–0.0208) compared to (0.0230–0.0308) for ${\text{W}}^{\left(5nn\right)}$ . Finally, we also investigated using non-binary versions of $\left\{{\text{W}}^{\left(O\right)},{\text{W}}^{\left(S\right)}\right\}$ , but these performed very poorly and the results are displayed in Section S4.2 of the Supplemental materials.

### 4.3. Pollution-health effects

The effect of each pollutant on each medication outcome is presented as a relative risk (RR), where a value of one corresponds to no association between air pollution concentrations and respiratory prescription rates. An RR greater than one indicates that increased concentrations are associated with an increased rate of respiratory prescriptions, while an RR less than one indicates a protective effect. Each relative risk presented here is computed for a one standard deviation increase in each pollutant, because this represents a realistic increase in the concentrations that could be observed in the present study. Specifically, these standard deviations are: NO_2_: 6.34 μ g/m ${}^{3}$ , PM_10_: 2.77 μ g/m ${}^{3}$ , and PM ${}_{2.5}$ : 1.68 μ g/m ${}^{3}$ . The relative risk results for NO_2_ and PM_10_ are displayed in Tables 1 and 2, respectively, while those for PM ${}_{2.5}$ are presented in Section S4.3 of the Supplemental materials. Each table presents the results from 18 model runs, which include all combinations of the three medication types, three neighbourhood matrices ( ${\text{W}}^{\left(5nn\right)}$ , ${\text{W}}^{\left(O\right)}$ and ${\text{W}}^{\left(S\right)}$ , the latter two using the optimal thresholds) and two time periods. The results for each model run comprise the posterior median relative risk and a 95% credible interval, the exceedance probability that the estimated relative risk is greater than one, and the Deviance Information Criterion (DIC) and Watanabe-Akaike Information Criterion (WAIC) model fit metrics.

**Table 1.** Posterior median relative risk estimates, 95% credible intervals and exceedance probabilities from models based on $\left\{{\text{W}}^{\left(5nn\right)},{\text{W}}^{\left(O\right)},{\text{W}}^{\left(S\right)}\right\}$ quantifying the impacts of NO_2_ (based on an increase of 6.34 μ g/m ${}^{3}$ ) on respiratory prescription rates for all three medication types and both study periods.

Medication	Matrix	Period	Threshold	RR	EP	95% CI	DIC	WAIC
Reliever	${\text{W}}^{\left(5nn\right)}$	2016–2020	–	0.9967	0.1150	(0.9912, 1.0021)	477,965.5	491,837.1
		2016–2019	–	1.0040	0.9189	(0.9984, 1.0097)	382,085.9	392,721.4
	${\text{W}}^{\left(O\right)}$	2016–2020	0.0001	0.9943	0.0054	(0.9900, 0.9987)	476,169.3	489,441.9
		2016–2019	0.0001	1.0012	0.6980	(0.9967, 1.0056)	381,224.5	390,015.6
	${\text{W}}^{\left(S\right)}$	2016–2020	0.05	0.9956	0.0271	(0.9912, 1.0001)	475,787.2	489,265.1
		2016–2019	0.05	1.0031	0.9094	(0.9986, 1.0076)	381,067.6	390,084.6
Preventer	${\text{W}}^{\left(5nn\right)}$	2016–2020	–	0.9974	0.1797	(0.9918, 1.0030)	437,902.3	448,002.2
		2016–2019	–	1.0033	0.8627	(0.9974, 1.0091)	350,102.6	356,375.1
	${\text{W}}^{\left(O\right)}$	2016–2020	0.0005	0.9994	0.4000	(0.9945, 1.0043)	438,203.7	448,005.4
		2016–2019	0.0005	1.0045	0.9574	(0.9994, 1.0096)	350,422.7	356,842.3
	${\text{W}}^{\left(S\right)}$	2016–2020	0.1	0.9999	0.4902	(0.9952, 1.0047)	438,041.6	447,854.9
		2016–2019	0.1	1.0053	0.9824	(1.0004, 1.0103)	350,226.7	356,665.0
Both	${\text{W}}^{\left(5nn\right)}$	2016–2020	–	0.9976	0.1678	(0.9926, 1.0025)	525,300.0	543,534.2
		2016–2019	–	1.0039	0.9317	(0.9988, 1.0091)	419,565.5	433,303.9
	${\text{W}}^{\left(O\right)}$	2016–2020	0.0005	0.9972	0.1063	(0.9929, 1.0016)	526,202.0	542,255.4
		2016–2019	0.0001	1.0029	0.9063	(0.9986, 1.0072)	417,564.3	436,106.4
	${\text{W}}^{\left(S\right)}$	2016–2020	0.01	0.9959	0.0278	(0.9917, 1.0001)	521,707.8	545,464.6
		2016–2019	0.01	1.0034	0.9582	(0.9995, 1.0073)	417,731.7	435,337.5

RR: relative risk; EP: exceedance probability; CI: credible interval.

The thresholds used for each of $\left\{{\text{W}}^{\left(O\right)},{\text{W}}^{\left(S\right)}\right\}$ are also given.

**Table 2.** Posterior median relative risk estimates, 95% credible intervals and exceedance probabilities from models based on $\left\{{\text{W}}^{\left(5nn\right)},{\text{W}}^{\left(O\right)},{\text{W}}^{\left(S\right)}\right\}$ quantifying the impacts of PM_10_ (based on an increase of 2.77 μ g/m ${}^{3}$ ) on respiratory prescription rates for all three medication types and both study periods.

Medication	Matrix	Period	Threshold	RR	EP	95% CI	DIC	WAIC
Reliever	${\text{W}}^{\left(5nn\right)}$	2016–2020	–	1.0042	0.9479	(0.9991, 1.0092)	477,974.7	491,835.1
		2016–2019	–	1.0031	0.9225	(0.9988, 1.0074)	382,073.1	392,574.9
	${\text{W}}^{\left(O\right)}$	2016–2020	0.0001	0.9990	0.3576	(0.9934, 1.0045)	476,083.9	489,553.2
		2016–2019	0.0001	1.0006	0.5876	(0.9956, 1.0055)	381,222.0	389,984.1
	${\text{W}}^{\left(S\right)}$	2016–2020	0.05	1.0042	0.9738	(1.0000, 1.0084)	475,780.4	489,327.3
		2016–2019	0.05	1.0030	0.9402	(0.9992, 1.0067)	381,064.9	390,094.5
Preventer	${\text{W}}^{\left(5nn\right)}$	2016–2020	–	1.0062	0.9942	(1.0014, 1.0110)	437,915.3	448,050.3
		2016–2019	–	1.0045	0.9812	(1.0003, 1.0087)	350,110.8	356,376.4
	${\text{W}}^{\left(O\right)}$	2016–2020	0.0005	1.0075	0.9997	(1.0033, 1.0118)	438,202.9	448,008.2
		2016–2019	0.0005	1.0054	0.9971	(1.0016, 1.0092)	350,430.4	356,814.9
	${\text{W}}^{\left(S\right)}$	2016–2020	0.1	1.0066	0.9991	(1.0024, 1.0107)	438,036.2	447,858.2
		2016–2019	0.1	1.0046	0.9926	(1.0009, 1.0084)	350,226.9	356,666.0
Both	${\text{W}}^{\left(5nn\right)}$	2016–2020	–	1.0042	0.9536	(0.9993, 1.0090)	525,307.9	543,525.7
		2016–2019	–	1.0031	0.9222	(0.9988, 1.0073)	419,567.1	433,306.3
	${\text{W}}^{\left(O\right)}$	2016–2020	0.0005	1.0063	0.9981	(1.0020, 1.0105)	526,212.8	542,237.1
		2016–2019	0.0001	1.0017	0.7632	(0.9970, 1.0063)	417,577.8	436,097.6
	${\text{W}}^{\left(S\right)}$	2016–2020	0.01	1.0041	0.9768	(1.0001, 1.0081)	521,715.1	545,465.9
		2016–2019	0.01	1.0025	0.9112	(0.9989, 1.0060)	417,706.1	435,341.8

RR: relative risk; EP: exceedance probability; CI: credible interval.

The thresholds used for each of $\left\{{\text{W}}^{\left(O\right)},{\text{W}}^{\left(S\right)}\right\}$ are also given.

Table 1 shows that NO_2_ does not exhibit a strong association with respiratory medication prescriptions, because all RR estimates are very close to one across all medication types and time periods, while all but one of the 95% credible intervals include the null RR of one. The table also shows that for the pre-pandemic period (2016–2019) all relative risks are above one, while for the full time period (2016–2020) they are all below one. While the absolute differences in these posterior median relative risk are small between the two time periods the exceedance probabilities are typically substantially different, ranging between 0.6980 and 0.9824 for 2016–2019 and between 0.0054 and 0.4902 for 2016–2020.

The results for PM_10_ are shown in Table 2, and demonstrate that there are significant effects of PM_10_ on preventer medications for all models and time periods as all of the 95% credible intervals are wholly greater than one. The posterior median relative risks for this pollutant–outcome pair range between 1.0045 and 1.0075, suggesting that if PM_10_ increases by around 2.77 μ g/m ${}^{3}$ then the rate of preventer medication usage will increase by around 0.5%. This significant effect is reinforced by the exceedance probabilities, which are all above 98%. In contrast, there are no significant effects of PM_10_ on reliever medications, as all of the 95% credible intervals contain the null relative risk of one. However, there is some evidence of a potential effect, as the exceedance probabilities range between 0.3576 and 0.9738. Moreover, considering both medications jointly shows results in the middle of the previous two as expected, with relative risks ranging between 1.0017 and 1.0063 and exceedance probabilities ranging between 0.7632 and 0.9981. But in this case only two of the six estimates are statistically significant at the 0.05 level. Comparing the two data spans shows that the relative risks for the entire time period (2016–2020) are generally larger than those for the shorter time period (2016–2019), which is the opposite result observed for NO_2_. However, these differences do not generally affect the statistical significance.

The results for PM ${}_{2.5}$ are shown in Section S4.3 of the Supplemental materials for brevity, and are similar but slightly weaker than those for PM_10_. Additionally, the Supplemental materials present two further analyses for PM_10_, focusing on lagged effects (see Section S4.4) and the incorporation of exposure uncertainty when estimating the health effects (see Section S4.5). Finally, the tables of results also display the DIC and WAIC model fit metrics for each neighbourhood matrix. The tables show that for the reliever and both medication types ${\text{W}}^{\left(O\right)}$ and ${\text{W}}^{\left(S\right)}$ generally outperform ${\text{W}}^{\left(5nn\right)}$ in terms of both DIC and WAIC, while for the preventer medication type the results are much closer and ${\text{W}}^{\left(5nn\right)}$ typically performs better. Thus, while our proposed neighbourhood matrices do not always provide the best model fit they do so in the majority of situations, which taken together with their improved modelling of spatial correlation suggests that they are a superior approach compared to using the five nearest neighbours method.

## 5. Discussion

This study has developed spatio-temporal models to quantify the effects of air pollution on the volume of medications prescribed to treat chronic respiratory ill health like asthma and COPD within a primary care setting, and applied the models in a new study based in Scotland. This study extends the work of Blangiardo et al.^13^ and Lee^14^ to a longer and more recent time period, while examining multiple respiratory medications (reliever, preventer and both) and pollutants (NO_2_, PM_10_ and PM ${}_{2.5}$ ) to provide a comprehensive assessment of how air pollution influences chronic respiratory ill health that is managed in a non-hospitalised setting. Additionally, this study estimates population-level pollution exposures based on where the GP surgery patient populations are likely to live, rather than simply estimating the concentration at each GP surgery itself.

From a methodological standpoint, this study adjusts existing spatial areal unit smoothing models for the situation where the spatial extents of the areal units (i.e. GP surgery catchment areas) are partially unknown and overlapping. The challenge here is how to determine the spatial adjacency between these areal units, and this study proposes novel spatial adjacency structures based on population overlap data obtained on a second set of areal units (i.e. data zones) that have known geographical boundaries. We propose two specifications here, which measure spatial closeness based on whether the patient populations from two GP surgeries occupy the same ( ${\text{W}}^{\left(O\right)}$ ) or the same/spatially neighbouring ( ${\text{W}}^{\left(S\right)}$ ) data zones. These neighbourhood matrices thus consider where each GP surgery’s patient population actually live, rather than just considering the exact locations of the surgeries themselves. As a result, these neighbourhood matrices are better able to capture spatial dependencies and prevent over-smoothing of the spatial random effects than a commonly used five nearest neighbours approach is, which is evidenced by lower MAI and GSAI values. For example, the MAI values reduce by between 20.99% and 48.63% when using ${\text{W}}^{\left(O\right)}$ or ${\text{W}}^{\left(S\right)}$ in the model compared with using ${\text{W}}^{\left(5nn\right)}$ . For other researchers modelling the type of data considered here, we recommend fitting models with both ${\text{W}}^{\left(O\right)}$ and ${\text{W}}^{\left(S\right)}$ initially, so that the consistency of the results can be observed. Here, the results are fairly consistent, but this may not always be the case. In practice, we recommend choosing between ${\text{W}}^{\left(O\right)}$ and ${\text{W}}^{\left(S\right)}$ using the MAI, as the role of the spatial random effects is to capture the unmeasured correlation in the data.

This study provides robust evidence that PM_10_ has a consistent significant association with prescriptions of medications used to prevent respiratory ill health events, as the exceedance probabilities exceed 98% across all spatial structures and both data spans (2016–2019 and 2016–2020). The posterior median relative risks suggest that the rate of prescriptions of such medications increases on average by 0.5% when PM_10_ increases by 2.77 $\mu {\text{g/m}}^{3}$ . Although this estimated effect size is small, the population at risk of air pollution is large. For example, over the study period about 2,002,720 items of this type of medication were prescribed on average across the entire country each year, which when multiplied by the above relative risk equates to an additional 10,014 items of preventer medication each year. In contrast, there are no consistent associations between medications used to relieve the symptoms of respiratory ill health and PM_10_, while the results when both medications are combined show weak evidence of an effect as their results are a compromise between those for the two medication types. Additionally, the effects of PM ${}_{2.5}$ on respiratory prescription rates are weaker than the corresponding effects of PM_10_, with smaller exceedance probabilities and posterior median relative risks in most cases. In contrast, across all models and medication types NO_2_ consistently demonstrates no association with prescription rates, with relative risk estimates being close to one and 95% credible intervals that almost always include one.

Comparing the two data spans highlights the potential influence of the Covid-19 pandemic on the relationship between air pollution and respiratory prescription rates. The most notable difference is observed for NO_2_, where the exceedance probabilities are substantially higher for the pre-pandemic period compared to the whole time period, while the corresponding relative risks are generally above one and below one respectively. That said, in all cases the associations are not significant at the 0.05 level. For PM_10_, the differences between the two data spans are smaller, with slightly weaker associations in the pre-pandemic period, although this does not alter the statistical significance of the findings. In contrast, PM ${}_{2.5}$ exhibits almost no differences, suggesting that its impact on prescriptions remained stable despite pandemic-related disruptions. There are a number of possible reasons for these pandemic-related differential health impacts, including changes in the lifestyle and healthcare-seeking behaviours which may have affected the respiratory prescription counts. For example, the spike in prescription rates in March 2020 (see Figure 1) suggests that people stocked up on medications just before the first national lockdown. Additionally, the pandemic led to a reduction in pollution levels as a result of national lockdowns and other travel restrictions that reduced vehicle and other emissions. For example, based on pollution predictions used in this study, the mean concentrations across Scotland in March 2020 reduced by 0.12 μ g/m ${}^{3}$ (NO_2_), 0.07 μ g/m ${}^{3}$ (PM_10_) and 0.16 μ g/m ${}^{3}$ (PM ${}_{2.5}$ ) compared to the corresponding concentrations for March 2019. These reductions were still evident later that year, as a result of the additional national and regional lockdowns imposed by the Scottish Government.

A limitation of this study is that the proposed approach to modelling the spatial correlation structure is crucially dependent on additional population distribution data that may not be available in all countries. For instance in developing countries like China, where primary healthcare is less structured, patients often visit multiple hospitals or pharmacies without adequate registration systems, making it challenging to construct a meaningful neighbourhood matrix based on GP registration data. A further limitation is that the spatio-temporal model used assumes that the length of the random effects vector and the neighbourhood structure captured by W remains constant throughout the 60-month study period, and thus does not dynamically adjust to potential changes in GP surgery locations or shifts in patient registration patterns over time. Therefore, in future work we will explore alternative random effects models with adaptive neighbourhood structures that can evolve over time to reflect changes in patient distribution and differing numbers of GP surgeries. The study also assumes that the spatial distribution of the set of registered patients from each GP surgery to the surrounding data zones matches the distribution of those subset of patients actually receiving respiratory related prescriptions. This assumption may lead to potential misclassification, since disease prevalence and thus prescription rates differ across data zones served by a GP surgery. In the future we will explore a more flexible Bayesian hierarchical modelling approach, which will allow the proportions of patients receiving prescriptions from a GP surgery in each data zone to be treated as an unknown random vector and modelled using a Dirichlet distribution, at a latent level. This random vector can be informed by the observed proportions of registered patients in each data zone, with the mean of the Dirichlet distribution depending on these proportions.

## Supplemental Material

sj-pdf-1-smm-10.1177_09622802261439259 - Supplemental material for Quantifying the effects of air pollution on respiratory ill health treated in primary care when the locations of the populations at risk are partially unknown Supplemental material, sj-pdf-1-smm-10.1177_09622802261439259 for Quantifying the effects of air pollution on respiratory ill health treated in primary care when the locations of the populations at risk are partially unknown by Qiangqiang Zhu, Duncan Lee and Oliver Stoner in Statistical Methods in Medical Research
