# Supplementary material for: Quantifying the effects of air pollution on respiratory ill health treated in primary care when the locations of the populations at risk are partially unknown
Source: Stat Methods Med Res. 2026 Apr 24;35(6):1215–29. doi: 10.1177/09622802261439259 (PMC13283498; doi:10.1177/09622802261439259)
Supplement: sj-pdf-1-smm-10.1177_09622802261439259 - Supplemental material for Quantifying the effects of air pollution on respiratory ill health treated in primary care when the locations of the populations at risk are partially unknown [file sj-pdf-1-smm-10.1177_09622802261439259.pdf]

Supplementary material for "Quantifying the effects of air pollution on respiratory ill health treated in primary care when the locations of the populations at risk are partially unknown"

Qiangqiang Zhu, Duncan Lee, and Oliver Stoner

This supplementary material includes additional descriptions and information not included in the main paper. Section S1 provides further details on the motivating study, while Section S2 describes the prior specification for the Bayesian spatio-temporal CAR-AR(1) models. Section S3 examines the thresholds applied to the neighbourhood matrices to mitigate negligible population intersections and assess robustness. Finally, Section S4 presents additional results from the motivating study that are not presented in the main paper for brevity.

S1 Additional details on the motivating study

S1.1 Further details of the prescription data

The Scotland-wide prevalence rates for asthma and COPD by age and sex group within the study period can be seen in Figure S1, which shows that prevalence is initially higher in males until adolescence, after which females exhibit higher rates. This sex difference in prevalence by age group is influenced by multiple factors, including socioeconomic status, nutrition, comorbidities, health care access and occupational exposures (Chowdhury et al., 2021). Additionally, prevalence declines in elderly people over 85, possibly due to misreporting or underdiagnosis of symptoms by the oldest groups or a "health survivor effect", where the oldest groups consist of comparatively healthier individuals, as those with severe asthma and COPD conditions may not have survived to this age (Delmas et al., 2021).

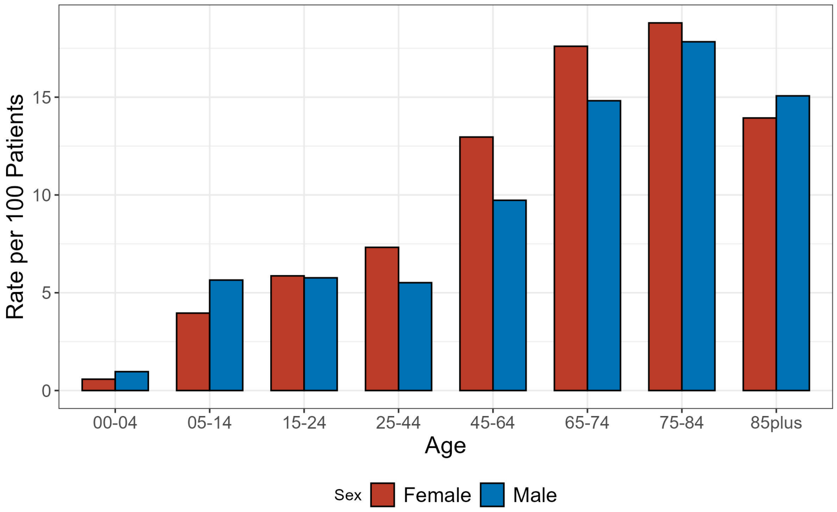

Figure S1: National average prevalence rates of asthma and COPD by age and sex in Scotland.

S1.2 Further description of the SIMD indicators

The Scottish index of multiple deprivation (SIMD) includes both count-based and rate-based indicators for similar quantities, and to simplify the analysis all count-type indicators are excluded in favour of their corresponding rate-based equivalents. The description of the remaining indicators in each domain is provided in Table S1, while Figure S2 presents histograms and density plots of their distributions. The figure shows that some indicators exhibit high skewness or outliers, with both *EduAttend* and *EduAttain* appearing to exhibit unusual zero values. The minimum values after excluding these zeros are 0.4 for *EduAttend* and 2.890 for *EduAttain*, suggesting that zeros might not represent the true education levels but rather indicate the absence of pupils or education records in certain Data Zones. Therefore, zeros in these two indicators are replaced with the minimum non-zero value, which represents the lowest level excluding areas with zero values.

Figure S3 shows a correlation plot of the 20 remaining SIMD indicators. Several of the indicators are highly correlated, with for example *IncRate*, *EmpRate*, and *EduNoQuals* exhibiting Pearson correlation coefficients exceeding 0.8. Similarly, indicators within the "Access to services" domain are also strongly correlated, and such strong multicollinearity between covariates can lead to imprecise effect estimation. To address this, we applied a variance inflation factor (VIF)-based selection procedure to the SIMD indicators, sequentially removing indicators with $\text{VIF} > 5$ until all remaining indicators are below this threshold. This results in a final set of 14 indicators for inclusion in the models.

Table S1: Summary of the indicators included in the 2020 Scottish Index of Multiple Deprivation (SIMD) that are used in this study.

Domain	Description	Type	Label
**Income**	Percentage of people who are income deprived.	Percentage	IncRate
**Employment**	Percentage of working age people who are employment deprived.	Percentage	EmpRate
**Education**	School pupil attendance.	Percentage	EduAttend
	Attainment of school leavers.	Score	EduAttain
	Working age people with no qualifications.	Standardised ratio	EduNoQuals
	Proportion of people aged 16-19 not participating in education, employment or training.	Proportion	EduPartici
	Proportion of 17-21 year olds entering university.	Proportion	EduUniver
**Access**	Average drive time to a petrol station.	Time (minutes)	GAccPetrol
	Average drive time to a GP surgery.	Time (minutes)	GAccDTGP
	Average drive time to a post office.	Time (minutes)	GACCDTPost
	Average drive time to a primary school.	Time (minutes)	GACCDTPsch
	Average drive time to a retail centre.	Time (minutes)	GAccDTRet
	Average drive time to a secondary school.	Time (minutes)	GAccDTSsch
	Public transport travel time to a GP surgery.	Time (minutes)	GAccPTGP
	Public transport travel time to a post office.	Time (minutes)	GAccPTPost
	Public transport travel time to a retail centre.	Time (minutes)	GAccPTRet
	Percentage of premises without access to superfast broadband (at least 30Mb/s download speed).	Percentage	GAccBrdbnd
**Crime**	Recorded crimes of violence, sexual offences, domestic housebreaking, vandalism, drugs offences, and common assault per 10,000 population.	Rate	CrimeRate
**Housing**	Percentage of people in overcrowded households.	Percentage	HouseOCrat
	Percentage of people in households without heating.	Percentage	HouseNCrat

S2 Prior specification for the Bayesian spatio-temporal model

A half-normal prior was specified for the precision parameter τ on the standard deviation scale (i.e., for $\sigma = 1/\sqrt{\tau}$) following the recommendation from Gelman (2006). Specifically, the probability density function of σ is given by

$$\sigma \sim \text{Half-Normal}(0, \sigma_0^2), \quad f_\sigma(\sigma|\sigma_0) = \frac{\sqrt{2}}{\sigma_0\sqrt{\pi}} \exp\left(-\frac{\sigma^2}{2\sigma_0^2}\right).$$

Here, larger values of σ allow greater uncertainty and hence variation in the spatio-temporal random effects, leading to greater variation in the disease risks $\{\theta_t(\mathcal{S}_k)\}$. There is no universally "correct" choice for σ_0, but extreme spatial variability is uncommon for disease risk on the natural log linear predictor scale. In this study σ_0 is set to 1.5,

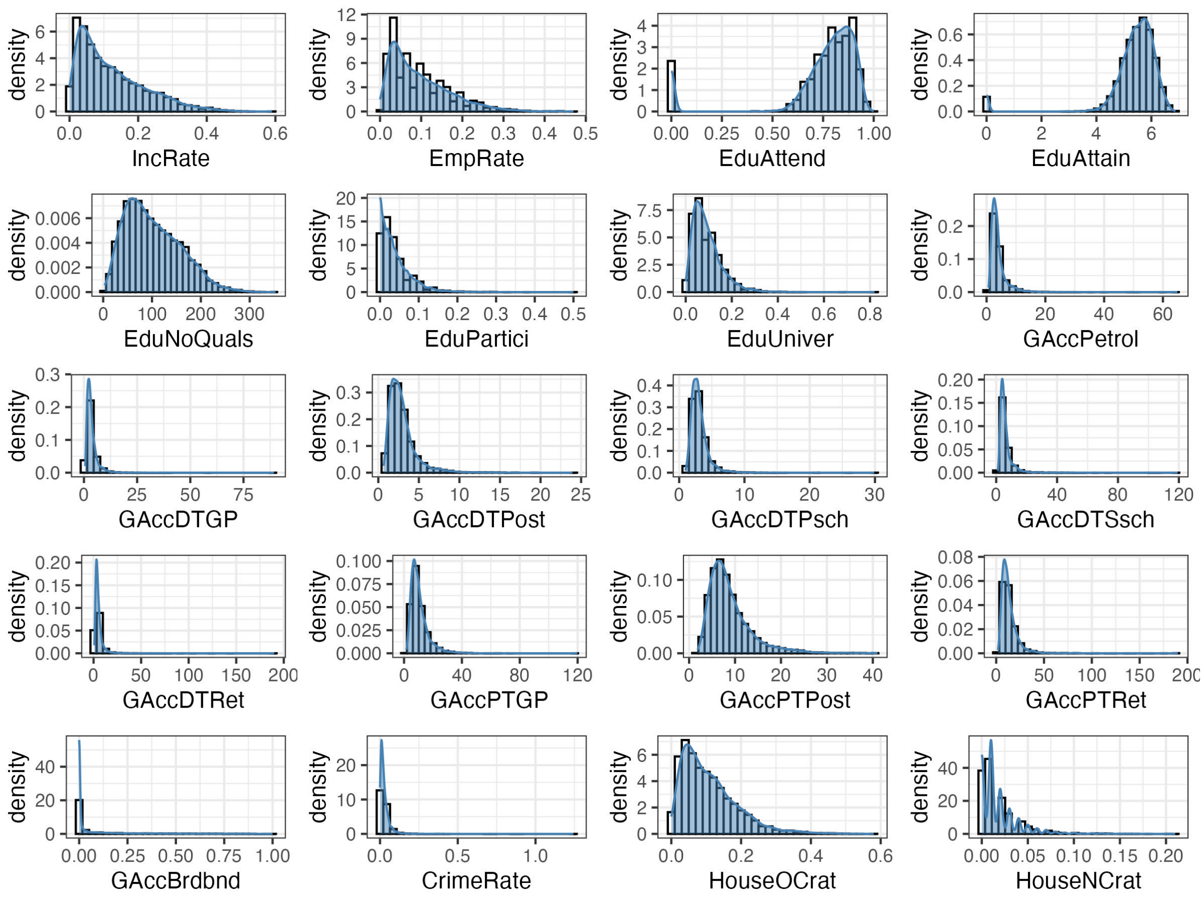

Figure S2: Histograms and density plots of the SIMD indicators at the Data Zone scale.

which offers flexibility to capture variation in log-relative risks while avoiding implausibly high values. As shown in Figure S4 (a), the prior density for σ drops sharply beyond 5, reflecting a low probability of extreme spatial variability. Within the `INLA` software a prior should be specified on the log precision scale, that is, $\theta = \log(\tau) = \log(1/\sigma^2)$. Using the change of variables formula the induced prior on the log precision θ is given by

$$\begin{aligned} f_\theta(\theta) &= f_\sigma\left\{\sigma = \exp(-\theta/2)\right\} \left|\frac{\partial\sigma}{\partial\theta}\right| \\ &= \frac{1}{\sqrt{2\pi\sigma_0^2}} \exp\left(-\frac{\theta}{2}\right) \exp\left(-\frac{\exp(-\theta)}{2\sigma_0^2}\right). \end{aligned}$$

Then taking a natural log transformation the log prior density is given by:

$$\ln\{f_\theta(\theta)\} = -\frac{1}{2}\ln(2\pi) - \ln(\sigma_0) - \frac{\theta}{2} - \frac{\exp(-\theta)}{2\sigma_0^2}.$$

For the spatial dependence parameter ρ a Gaussian prior with mean μ_γ and variance σ_γ^2 is assigned as the prior distribution on the logit scale $\gamma = \log(\frac{\rho}{1-\rho})$ by the `INLA`

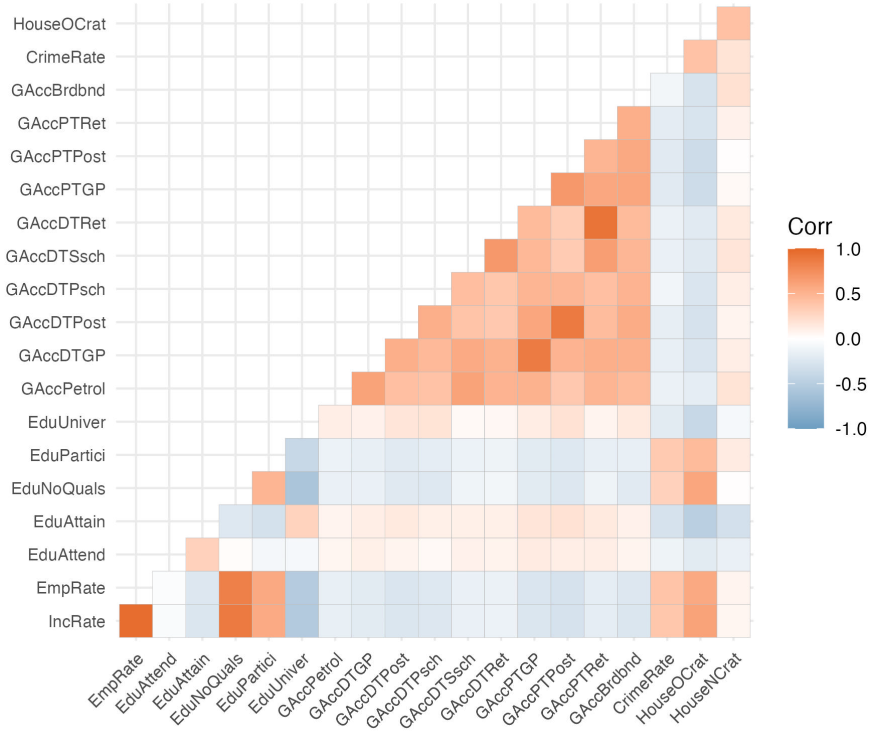

Figure S3: Correlation plot of the SIMD indicators at the Data Zone scale.

software, ensuring that ρ remains bounded between 0 and 1. The corresponding pdf of ρ is again obtained from the change of variables formula by

$$\begin{aligned}\gamma &= \log\left(\frac{\rho}{1-\rho}\right) \sim \mathrm{N}(\mu_\gamma, \sigma_\gamma^2),\\ f_\rho(\rho) &= f_\gamma\left\{\gamma = \log\left(\frac{\rho}{1-\rho}\right)\right\}\left|\frac{\partial\gamma}{\partial\rho}\right|\\ &= \frac{1}{\sqrt{2\pi}\sigma_\gamma}\exp\left(-\frac{\left(\log\left(\frac{\rho}{1-\rho}\right)-\mu_\gamma\right)^2}{2\sigma_\gamma^2}\right)\frac{1}{\rho(1-\rho)}.\end{aligned}$$

Here we specify $\mu_\gamma = 0$ and $\sigma_\gamma = 2$, and Figure S4 (b) presents the probability density curve for ρ. The resulting curve is weakly informative and exhibits a slight U-shape, with a higher density at the extremes near zero and one. The chosen prior does not impose strong assumptions on the distribution of ρ, allowing the data itself to exert more influence on estimating ρ. Finally, the temporal correlation parameter α is also assigned a Gaussian prior with mean μ_η and variance σ_η^2 on a transformed scale, this time on the $\eta = \log(\frac{1+\alpha}{1-\alpha})$ scale specified by the `INLA` software. This transformation and

the subsequent normal distribution ensure that α stays within the bounds of -1 and 1, maintaining the feasibility of the correlation parameter. Specifically,

$$
\begin{aligned}
\eta &= \log\left(\frac{1+\alpha}{1-\alpha}\right) \sim \mathrm{N}(\mu_\eta, \sigma_\eta^2), \\
f_\eta(\eta) &= \frac{1}{\sqrt{2\pi}\sigma_\eta} \exp\left(-\frac{\left(\log\left(\frac{1+\alpha}{1-\alpha}\right) - \mu_\eta\right)^2}{2\sigma_\eta^2}\right) \frac{2}{1-\alpha^2}.
\end{aligned}
$$

Here we specify $\mu_\eta = 0.25$ and $\sigma_\eta = 2$, and the pdf curve of α is given in Figure S4 (c). This prior is hence fairly weakly informative.

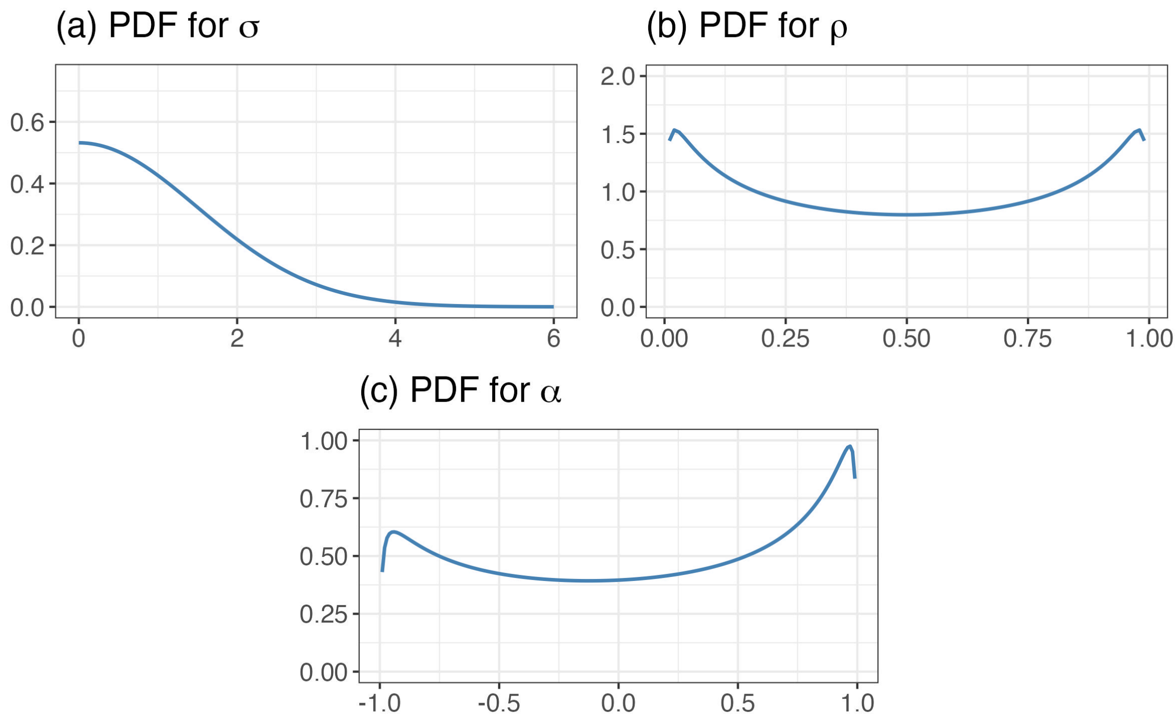

Figure S4: Probability density function (PDF) curves for the prior distributions assumed for the following parameters: (a) the standard deviation σ; (b) the spatial dependence ρ; and (c) the temporal correlation α.

S3 Choice of threshold for the neighbourhood matrices

The neighbourhood matrices $\mathbf{W}^{(O)}$ and $\mathbf{W}^{(S)}$ proposed in Section 3.3 have thresholding applied to them, whereby very small values of $(w_{kj}^{*(O)}, w_{kj}^{*(S)})$ are set to zero to re-

move the influence of negligible population overlaps. The thresholds considered are $\varepsilon \in \{0.0001, 0.0005, 0.001, 0.005, 0.01\}$ for $\mathbf{W}^{(O)}$ and $\alpha \in \{0.005, 0.01, 0.05, 0.1, 0.5\}$ for $\mathbf{W}^{(S)}$, which are different because the elements $w_{kj}^{*(S)}$ are typically larger than the corresponding elements $w_{kj}^{*(O)}$ because of the additional spatial overlaps considered in the construction of the former. Tables S2 and S3 provide summary statistics for the distribution of the numbers of neighbours (that is $w_{kj}^{(O)} = 1$ or $w_{kj}^{(S)} = 1$) that each GP surgery has at different threshold values in the neighbourhood matrices $\mathbf{W}^{(O)}$ and $\mathbf{W}^{(S)}$ respectively.

When $\varepsilon = 0$ and $\alpha = 0$, which means no threshold is applied, each GP surgery has on average over 100 spatial connections with other GP surgeries. This suggests a highly interconnected network where a substantial proportion of GPs are considered neighbours of each other, which goes against the notion of a neighbourhood matrix capturing local spatial proximity. This high connectivity may lead to over-smoothing, given the induced correlations from the CAR model between GP surgeries that are actually far apart. On the other hand, the maximum threshold values of $\{\varepsilon, \alpha\}$ result in only 3 or 4 connections on average for each GP surgery, meaning that some spatially close GP surgeries will be treated as conditionally independent in the modelling. Intermediate values of $\{\varepsilon, \alpha\}$ thus seem to offer a more reasonable compromise between connectivity and sparsity, but as discussed in the main paper, the optimal values will be chosen as the ones that minimise the level of residual spatial correlation.

Table S2: Summary statistics for the number of neighbours for each GP surgery in the binary matrix $\mathbf{W}^{(O)}$ at different threshold values of ε for the data spans 2016–2020 and 2016–2019.

	2016–2020						2016–2019					
ε	0	0.0001	0.0005	0.001	0.005	0.01	0	0.0001	0.0005	0.001	0.005	0.01
Min.	5	1	1	1	1	1	3	1	1	1	1	1
1st Qu.	63	10	6	5	3	2	57	10	6	5	2	1
Median	110	17	12	10	5	3	101	17	12	10	5	3
Mean	135	27	17	13	6	3	125	27	17	13	6	3
3rd Qu.	199	30	23	18	9	5	183	31	23	18	8	5
Max	419	159	85	61	20	13	384	157	84	63	20	13

S4 Additional results from the motivating study

This section presents a number of additional results from the motivating study that are not included in the paper due to space constraints.

Table S3: Summary statistics for the number of neighbours for each GP surgery in the binary matrix $\mathbf{W}^{(S)}$ at different threshold values of α for the data spans 2016–2020 and 2016–2019.

	2016–2020						2016–2019					
α	0	0.005	0.01	0.05	0.1	0.5	0	0.005	0.01	0.05	0.1	0.5
Min.	6	1	1	1	1	1	3	1	1	1	1	1
1st Qu.	137	23	19	12	9	2	123	22	19	12	9	2
Median	241	33	28	18	13	4	214	32	28	18	13	4
Mean	245	42	35	20	15	4	225	41	35	20	15	4
3rd Qu.	356	56	48	26	20	6	332	56	47	26	20	6
Max	613	177	139	67	44	16	580	176	138	67	44	16

S4.1 Residual correlation analysis from using the $\mathbf{W}^{(S)}$ neighbourhood matrix

Figure S5 presents Moran's I statistics for the monthly residuals from the spatio-temporal CAR-AR(1) model based on $\mathbf{W}^{(S)}$, which is fitted to the data using the five thresholds $\alpha = \{0.005, 0.01, 0.05, 0.1, 0.5\}$ listed in the main paper. The figure has the same format as that for $\mathbf{W}^{(O)}$ presented in the main paper, and shows that the biggest threshold $\alpha = 0.5$ leads to the highest MAI values, indicating that using this value results in substantial spatial correlation being present in the residuals. In contrast, moderate thresholds such as $\alpha \in \{0.01, 0.05, 0.1\}$ better capture the residual spatial dependencies in the different data sets, as evidenced by lower PSAI, GSAI and MAI values.

S4.2 Residual correlation analysis from using non-binary versions of the $\{\mathbf{W}^{(O)}, \mathbf{W}^{(S)}\}$ neighbourhood matrices

In initial model development we used non-binary versions of $\{\mathbf{W}^{(O)}, \mathbf{W}^{(S)}\}$, because we thought that this would better represent the population overlaps between GP surgeries and hence the spatial correlation structure in the data. These non-binary matrices were constructed from Equations (3) and (4) in the main paper by

$$w_{kj}^{(O)} = \begin{cases} w_{kj}^{*(O)} & \text{if } w_{kj}^{*(O)} > \varepsilon \text{ and } k \neq j \\ 0 & \text{otherwise} \end{cases}, \quad w_{kj}^{(S)} = \begin{cases} w_{kj}^{*(S)} & \text{if } w_{kj}^{*(S)} > \alpha \text{ and } k \neq j \\ 0 & \text{otherwise} \end{cases},$$

which is analogous to Equation (5) in the main paper except that the non-zero values are not fixed at one. When fitting the spatio-temporal CAR-AR(1) model using these

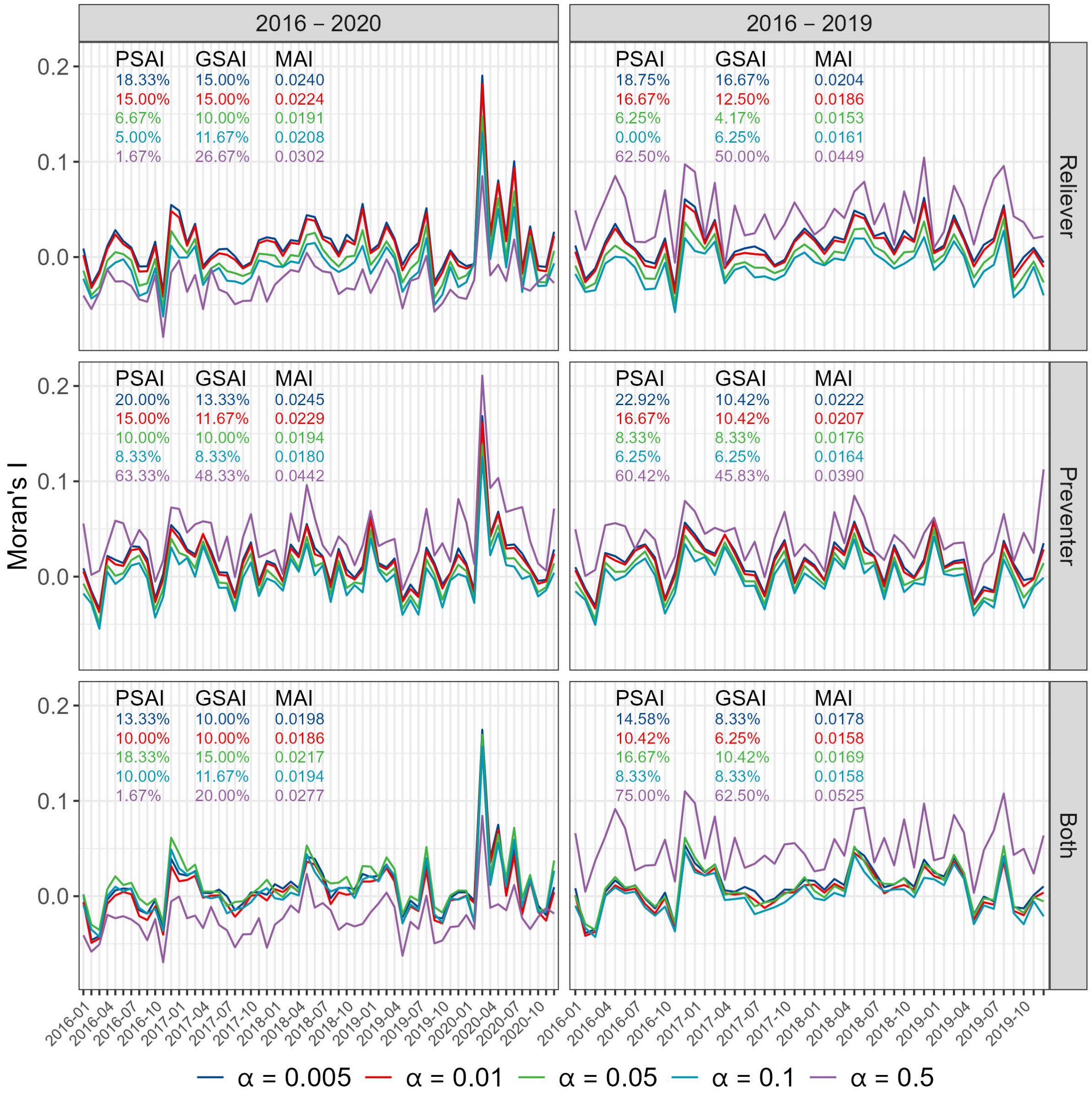

Figure S5: Moran's I statistics quantifying the level of spatial correlation in the residuals for each month when using $\mathbf{W}^{(S)}$ as the neighbourhood matrix. Each panel represents a different disease outcome and time period, while the coloured lines relate to the different thresholds α. PSAI, GSAI and MAI indicators are listed in each case.

non-binary neighbourhood matrices we fixed $\rho = 1$, which corresponds to the intrinsic CAR prior proposed by Besag et al. (1991). The reason for this is that when fitting the CAR prior proposed by Leroux et al. (2000) using the `R` package `INLA` we used the `besagproper2` latent model, but this only fits binary matrices. In contrast, the intrinsic CAR prior can be fitted with non-binary neighbourhood matrices using the `generic0` latent model, and as most values of ρ were very close to one when fitting the binary neighbourhood matrices this restriction will have little impact on the results.

The levels of residual spatial correlation from using these non-binary neighbourhood matrices in the model are summarised in Figures S6 and S7, which relate to $\mathbf{W}^{(O)}$ and $\mathbf{W}^{(S)}$ respectively. Compared with their binary counterparts the non-binary matrices lead to consistently higher PSAI, GSAI, and MAI values across almost all threshold settings. For example, when modelling both medication types between 2016 and 2019 using $\mathbf{W}^{(O)}$, the lowest MAI values over the five thresholds are 0.0162 for the binary matrix and 0.0951 for the non-binary one. This indicates that the binary specifications are better able to capture the spatial correlation structure amongst the GP surgery prescription counts, and are hence used when quantifying the effects of each air pollutant on each disease outcome in the main paper.

S4.3 Relative risk results for $PM_{2.5}$

Table S4 presents the relative risk results for $PM_{2.5}$, which are in the same format as the NO_2 and PM_{10} results presented in the main paper. The table shows significant effects of $PM_{2.5}$ on preventer medications, with three of the six 95% credible intervals being equal to or above the null relative risk of one, as well as exceedance probabilities ranging between 0.801 and 0.985. However, the sizes of the posterior median relative risks are less than those from the corresponding models for PM_{10}, indicating that a one standard deviation increase in $PM_{2.5}$ concentrations would lead to a smaller percentage increase in prescription rates for preventing chronic respiratory diseases. For the reliever and both medication types there are no significant effects, as all of the 95% credible intervals contain the null relative risk of one.

S4.4 Results for PM_{10} lagged by one month

To explore the potential delayed effects of air pollution we refitted the models using PM_{10} concentrations lagged by one month, with this pollutant being chosen because it is the one that exhibits the strongest relationships with respiratory prescription rates. Table S5 summarises the posterior median relative risks, exceedance probabilities and 95% credible intervals from the lag-1 analysis, which can be compared to the non-lagged (lag-0) results presented in Table 2 in the main paper. The tables show that at lag-1 the PM_{10} relative risks are smaller than those obtained using lag-0. For example, between 2016–2020 the estimated RRs for preventer prescriptions using $\mathbf{W}^{(O)}$ decrease from 1.0075 (95% CI:

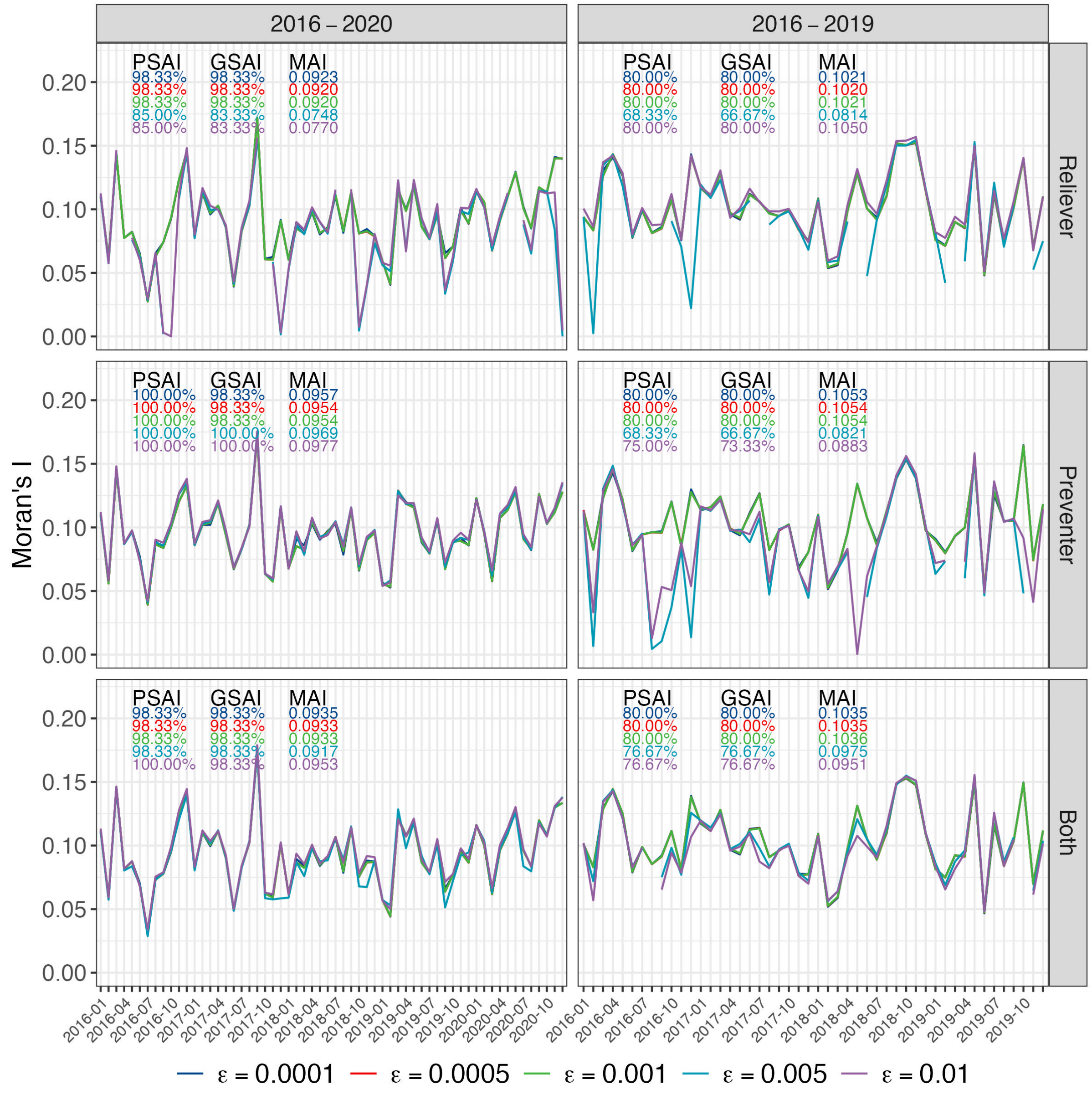

Figure S6: Moran's I statistics quantifying the level of spatial correlation in the residuals for each month when using a non-binary version of $\mathbf{W}^{(O)}$ as the neighbourhood matrix. Each panel represents a different disease outcome and time period, while the coloured lines relate to the different thresholds ε. PSAI, GSAI and MAI indicators are listed in each case.

1.0033–1.0118) at lag-0 to 1.0015 (95% CI: 1.0004–1.0026) at lag-1. Similar attenuation is observed across other medication types and spatial structures. However, there is still evidence that PM_{10} is significantly associated with preventer medication at lag-1, with three of the 95% credible intervals being equal to or above the null RR of one.

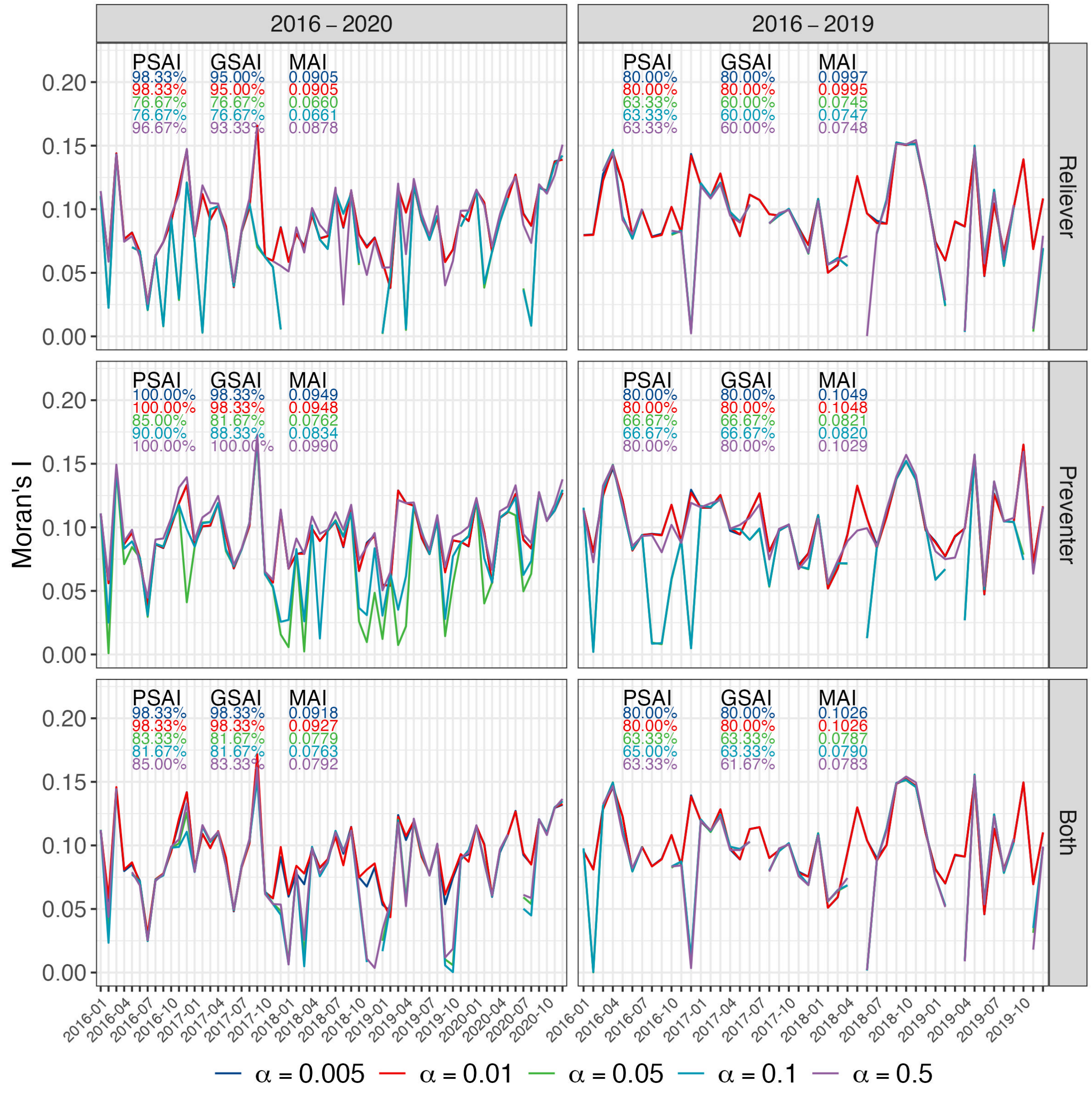

Figure S7: Moran's I statistics quantifying the level of spatial correlation in the residuals for each month when using a non-binary version of $\mathbf{W}^{(S)}$ as the neighbourhood matrix. Each panel represents a different disease outcome and time period, while the coloured lines relate to the different thresholds α. PSAI, GSAI and MAI indicators are listed in each case.

S4.5 Results for PM_{10} incorporating exposure uncertainty

Here, we assess the influence of prediction uncertainty in the modelled pollution concentrations on the estimated pollution-health associations. Our focus is on the association between PM_{10} and prescriptions of preventer medications, because this is the pollutant-outcome pair that exhibits a consistent significant association in the results presented in the main paper. To quantify the uncertainty in the predicted PM_{10} concentrations we

Table S4: Posterior median relative risk estimates, 95% credible intervals and exceedance probabilities from models based on $\{\mathbf{W}^{(5nn)}, \mathbf{W}^{(O)}, \mathbf{W}^{(S)}\}$ quantifying the impacts of $PM_{2.5}$ (based on an increase of 1.68 $\mu g/m^3$) on respiratory prescription rates for all three medication types and both study periods. The thresholds used for each of $\{\mathbf{W}^{(O)}, \mathbf{W}^{(S)}\}$ are also given.

Medication	Matrix	Period	Threshold	RR	EP	95% CI	DIC	WAIC
Reliever	$\mathbf{W}^{(5nn)}$	2016–2020	–	1.0010	0.7101	(0.9974, 1.0047)	477970.1	491848.2
		2016–2019	–	1.0019	0.8689	(0.9986, 1.0051)	382087.8	392730.3
	$\mathbf{W}^{(O)}$	2016–2020	0.0001	1.0001	0.5176	(0.9966, 1.0035)	476096.6	489527.8
		2016–2019	0.0001	1.0008	0.6773	(0.9975, 1.0040)	381213.3	390015.4
	$\mathbf{W}^{(S)}$	2016–2020	0.05	1.0019	0.8958	(0.9989, 1.0049)	475781.5	489325.6
		2016–2019	0.05	1.0023	0.9517	(0.9996, 1.0050)	381067.5	390072.2
Preventer	$\mathbf{W}^{(5nn)}$	2016–2020	–	1.0016	0.8011	(0.9980, 1.0051)	437906.1	448018.1
		2016–2019	–	1.0021	0.8884	(0.9987, 1.0053)	350103.3	356373.0
	$\mathbf{W}^{(O)}$	2016–2020	0.0005	1.0035	0.9823	(1.0002, 1.0068)	438202.8	448008.5
		2016–2019	0.0005	1.0033	0.9851	(1.0003, 1.0063)	350427.0	356812.3
	$\mathbf{W}^{(S)}$	2016–2020	0.1	1.0030	0.9728	(0.9999, 1.0061)	438038.9	447859.5
		2016–2019	0.1	1.0029	0.9755	(1.0000, 1.0057)	350226.3	356669.1
Both	$\mathbf{W}^{(5nn)}$	2016–2020	–	1.0004	0.5835	(0.9969, 1.0038)	525302.4	543533.5
		2016–2019	–	1.0012	0.7639	(0.9980, 1.0043)	419566.2	433312.6
	$\mathbf{W}^{(O)}$	2016–2020	0.0005	1.0024	0.9331	(0.9993, 1.0055)	526211.4	542228.1
		2016–2019	0.0001	1.0006	0.6423	(0.9975, 1.0036)	417560.9	436085.0
	$\mathbf{W}^{(S)}$	2016–2020	0.01	1.0017	0.8813	(0.9989, 1.0045)	521701.3	545480.6
		2016–2019	0.01	1.0018	0.9214	(0.9993, 1.0043)	417695.3	435350.6

simulate 100 draws from its predictive distribution. As described in Zhu et al. (2024), the best prediction model for PM_{10} is a linear regression model fitted on the natural log scale. One hundred sets of predicted pollution surfaces are sampled from this predictive distribution, and are subsequently back transformed to the original (un-logged) scale.

To quantify the impact that propagating pollution uncertainty into the health model has on the estimated pollution-health association, we follow the approach proposed by Blangiardo et al. (2016) who also modelled prescription rate data. Specifically, we re-fit the spatio-temporal model for each of the 100 simulated PM_{10} surfaces, and generate 1,000 posterior samples of the PM_{10} association for each model run. This results in 100,000 posterior samples of the pollution-health association, which allow for the uncertainty in the predicted PM_{10} concentrations. The results are presented in Table S6 for both study time periods and the three neighbourhood matrices. The statistical significance of the relative risks remains unchanged after accounting for prediction uncertainty, because all six of the 95% credible intervals are wholly above the null risk of one. However, the posterior median relative risks are slightly bigger in magnitude, ranging between 1.0073

Table S5: Posterior median relative risk estimates, 95% credible intervals and exceedance probabilities from models based on $\{\mathbf{W}^{(5nn)}, \mathbf{W}^{(O)}, \mathbf{W}^{(S)}\}$ quantifying the impacts of Lag-1 PM_{10} (based on an increase of 2.77 $\mu g/m^3$) on respiratory prescription rates for all 3 medication types and both study periods. The thresholds used for each of $\{\mathbf{W}^{(O)}, \mathbf{W}^{(S)}\}$ are also given.

Medication	Matrix	Period	Threshold	RR	EP	95% CI	DIC	WAIC
Reliever	$\mathbf{W}^{(5nn)}$	2016–2020	–	1.0014	0.9929	(1.0003, 1.0025)	481256.8	489984.7
		2016–2019	–	0.9984	0.0019	(0.9974, 0.9995)	380160.9	387005.9
	$\mathbf{W}^{(O)}$	2016–2020	0.0001	1.0017	0.9999	(1.0008, 1.0026)	479224.6	493091.0
		2016–2019	0.0005	0.9985	0.0009	(0.9976, 0.9995)	378583.3	386839.0
	$\mathbf{W}^{(S)}$	2016–2020	0.01	1.0000	0.4730	(0.9990, 1.0009)	475280.9	489056.2
		2016–2019	0.1	0.9985	0.0008	(0.9976, 0.9994)	378266.5	386998.4
Preventer	$\mathbf{W}^{(5nn)}$	2016–2020	–	1.0016	0.9977	(1.0005, 1.0027)	436697.5	444753.8
		2016–2019	–	0.9977	0.0000	(0.9966, 0.9989)	345821.3	350250.8
	$\mathbf{W}^{(O)}$	2016–2020	0.0001	1.0015	0.9955	(1.0004, 1.0026)	436944.8	445331.7
		2016–2019	0.005	0.9977	0.0000	(0.9966, 0.9988)	346111.8	350060.6
	$\mathbf{W}^{(S)}$	2016–2020	0.05	1.0015	0.9963	(1.0004, 1.0026)	436995.3	445833.3
		2016–2019	0.05	0.9977	0.0000	(0.9966, 0.9988)	345954.3	350779.6
Both	$\mathbf{W}^{(5nn)}$	2016–2020	–	1.0012	0.9882	(1.0002, 1.0023)	528234.6	537449.0
		2016–2019	–	0.9979	0.0000	(0.9969, 0.9989)	417530.0	426135.5
	$\mathbf{W}^{(O)}$	2016–2020	0.005	1.0012	0.9885	(1.0002, 1.0023)	528699.8	536190.5
		2016–2019	0.005	0.9979	0.0000	(0.9969, 0.9989)	417709.6	426737.8
	$\mathbf{W}^{(S)}$	2016–2020	0.005	0.9997	0.2387	(0.9989, 1.0005)	522955.6	548082.5
		2016–2019	0.01	0.9981	0.0000	(0.9974, 0.9989)	416726.9	433938.9

and 1.0094 which compares to a range of 1.0045 to 1.0075 when pollution uncertainty is ignored. Thus, while the absolute size of the relative risks has changed slightly, their statistical significance and hence the main conclusion from the study have not changed.

Table S6: Posterior median relative risk estimates, 95% credible intervals and exceedance probabilities from models quantifying the effect of PM_{10} (based on an increase of 2.77 $\mu g/m^3$) on preventer medications while incorporating uncertainty in the PM_{10} concentrations.

Matrix	Period	Threshold	RR	EP	95% CI
$\mathbf{W}^{(5nn)}$	2016–2020	-	1.0094	1	(1.0075, 1.0117)
	2016–2019	-	1.0073	1	(1.0055, 1.0095)
$\mathbf{W}^{(O)}$	2016–2020	0.0005	1.0092	1	(1.0073, 1.0114)
	2016–2019	0.0005	1.0073	1	(1.0055, 1.0094)
$\mathbf{W}^{(S)}$	2016–2020	0.1	1.0093	1	(1.0074, 1.0115)
	2016–2019	0.1	1.0073	1	(1.0055, 1.0094)

References

Besag, J., York, J., and Mollié, A. (1991). Bayesian image restoration with two applications in spatial statistics. *Annals of the Institute of Statistics and Mathematics*, 43:1–59.

Blangiardo, M., Finazzi, F., and Cameletti, M. (2016). Two-stage Bayesian model to evaluate the effect of air pollution on chronic respiratory diseases using drug prescriptions. *Spatial and Spatio-temporal Epidemiology*, 18:1–12.

Chowdhury, N. U., Guntur, V. P., Newcomb, D. C., and Wechsler, M. E. (2021). Sex and gender in asthma. *European Respiratory Review*, 30(162).

Delmas, M.-C., Bénézet, L., Ribet, C., Iwatsubo, Y., Zins, M., Nadif, R., Roche, N., and Leynaert, B. (2021). Underdiagnosis of obstructive lung disease: findings from the french constances cohort. *BMC Pulmonary Medicine*, 21:1–10.

Gelman, A. (2006). Prior distributions for variance parameters in hierarchical models (comment on article by Browne and Draper). *Bayesian Analysis*, 1(3):515–534.

Leroux, B., Lei, X., and Breslow, N. (2000). *Estimation of Disease Rates in Small Areas: A New Mixed Model for Spatial Dependence*, chapter Statistical Models in Epidemiology, the Environment and Clinical Trials, Halloran, M and Berry, D (eds), pages 135–178. Springer-Verlag, New York.

Zhu, Q., Lee, D., and Stoner, O. (2024). A comparison of statistical and machine learning models for spatio-temporal prediction of ambient air pollutant concentrations in Scotland. *Environmental and Ecological Statistics*, 31(4):1085–1108.
